# TWEAK-Fn14 Axis Induces Calcium-Associated Autophagy and Cell Death To Control Mycobacterial Survival in Macrophages

**DOI:** 10.1128/spectrum.03172-22

**Published:** 2022-11-02

**Authors:** Yi-Ming Chen, Po-Yu Liu, Kuo-Tung Tang, Hung-Jen Liu, Tsai-Ling Liao

**Affiliations:** a Department of Medical Research, Taichung Veterans General Hospital, Taichung, Taiwan, Republic of China; b Rong Hsing Research Center for Translational Medicine, National Chung Hsing University, Taichung, Taiwan, Republic of China; c Ph.D. Program in Translational Medicine, National Chung Hsing University, Taichung, Taiwan, Republic of China; d Division of Allergy, Immunology and Rheumatology, Department of Internal Medicine, Taichung Veterans General Hospital, Taichung, Taiwan, Republic of China; e Division of Infection, Department of Internal Medicine, Taichung Veterans General Hospital, Taichung, Taiwan, Republic of China; f Institute of Molecular Biology, National Chung Hsing University, Taichung, Taiwan, Republic of China; g The iEGG and Animal Biotechnology Center, National Chung Hsing University, Taichung, Taiwan, Republic of China; University of Hawaii at Manoa

**Keywords:** TWEAK-Fn14 signaling, autophagy, calcium influx, cell death, tuberculosis

## Abstract

Autophagy is a natural defense mechanism that protects the host against pathogens. We previously demonstrated that mycobacterial infection upregulated tumor necrosis factor-like weak inducer of apoptosis (TWEAK) to promote autophagy and mycobacterial autophagosome maturation through activation of AMP-activated protein kinase (AMPK). Fibroblast growth factor-inducible 14 (Fn14) is the receptor of TWEAK. But the role of Fn14 in mycobacterial infection remains elusive. Herein, we observed increased expression of Fn14 in peripheral blood mononuclear cells of active tuberculosis (TB) patients. Downregulation of cellular Fn14 enhanced mycobacterial survival in macrophages. Conversely, Fn14 overexpression inhibited mycobacterial growth, suggesting that Fn14 can inhibit mycobacterial infection. The *in vitro* results revealed that TWEAK-promoted mycobacterial phagosome maturation is Fn14-dependent. We demonstrated that TWEAK-Fn14 signaling promotes oxidative stress to enhance the expression of stromal interaction molecule 1 (STIM1) and its activation of the Ca^2+^ channel ORAI1. Elevated calcium influx stimulated the activation of CaMCCK2 (calcium/calmodulin-dependent protein kinase kinase 2) and its downstream effector AMPK, thus inducing autophagy in early infection. Persistently TWEAK-Fn14 signaling caused cell death in late infection by reducing mitochondrial membrane potential, leading to mitochondrial ROS accumulation, and activating cell death-associated proteins. Genetic Fn14 deficiency or TWEAK blockers decreased oxidative stress-induced calcium influx, thus suppressing autophagy and cell death in mycobacteria-infected macrophages, and resulting in elevated mycobacterial survival. We propose that the TWEAK-Fn14 axis and calcium influx could be manipulated for anti-TB therapeutic purposes. Our results offer a new molecular machinery to understand the association between the TWEAK-Fn14 axis, calcium influx, and mycobacterial infection.

**IMPORTANCE** Tuberculosis remains a major cause of morbidity and mortality worldwide. We previously demonstrated a relationship between TWEAK and activation of the autophagic machinery, which promotes anti-mycobacterial immunity. The TWEAK-Fn14 axis is multi-functional and involved in the pathogenesis of many diseases, thus blockade of TWEAK-Fn14 axis has been considered as a potential therapeutic target. Here, we demonstrated that the TWEAK-Fn14 axis plays a novel role in anti-mycobacterial infection by regulating calcium-associated autophagy. Persistently, TWEAK-Fn14 signaling caused cell death in late infection by reducing mitochondrial membrane potential, leading to mitochondrial ROS accumulation, and activating cell death-associated proteins. TWEAK blocker or Fn14 deficiency could suppress oxidative stress and calcium-associated autophagy, resulting in elevated mycobacterial survival. We propose that the TWEAK-Fn14 axis and calcium influx could be manipulated for anti-TB therapeutic purposes. This study offers a new molecular machinery to understand the association between the TWEAK-Fn14 axis, calcium influx, and mycobacterial infection.

## INTRODUCTION

Tuberculosis (TB) is a major cause of morbidity and mortality worldwide. Autophagy is a well-conserved lysosomal degradation pathway that plays a key role in the innate defense mechanism against Mycobacterium (1), and is modulated by proinflammatory cytokines (2). Tumor necrosis factor (TNF) α mediates inflammation in response to Mycobacterium tuberculosis (3), but is also a key factor in the pathogenesis of rheumatoid arthritis and other autoimmune diseases (4). Accumulating evidence has shown that an elevated risk of latent TB infection (LTBI) reactivation and TB infection occurred in rheumatic patients receiving anti-TNF-α therapy (5, 6). Tumor necrosis factor-like weak inducer of apoptosis (TWEAK) is a TNF ligand superfamily member that mediates pleiotropic effects on a variety of cells via its receptor, fibroblast growth factor-inducible 14 (Fn14), and is mainly expressed in myeloid and immune cells (7). Increasing evidence has demonstrated that the TWEAK-Fn14 signaling pathway is implicated in several cell functions (8–11). But the role of TWEAK-Fn14 in infection is poorly understood. Previously, we demonstrated that TWEAK-induced autophagy and promoted mycobacterial autophagosome maturation through activation of AMP-activated protein kinase (AMPK) (12). After achieving LTBI status, increased intracellular microRNA-889 inhibited TWEAK expression to maintain mycobacterial survival (12). But the role of Fn14 in tuberculosis is uncertain.

Moreover, previous studies have observed that TWEAK and Fn14 are abundantly expressed in patients with rheumatic diseases, including rheumatoid arthritis, psoriatic arthritis, and systemic lupus erythematosus (13, 14). This raises the possibility that blocking TWEAK-Fn14 signaling may be of therapeutic benefit in inflammatory status (15). An elevated risk of TB was found in patients receiving anti-TNF-α therapy, but the effect of TWEAK-Fn14 blockade on TB diseases is unclear. In this study, we sought to investigate the role of Fn14 and dissect the regulatory mechanism of the TWEAK-Fn14 axis in control mycobacterial infection.

## RESULTS

### Increased levels of TWEAK secretion and Fn14 expression in TB patients.

**FIG 1:**
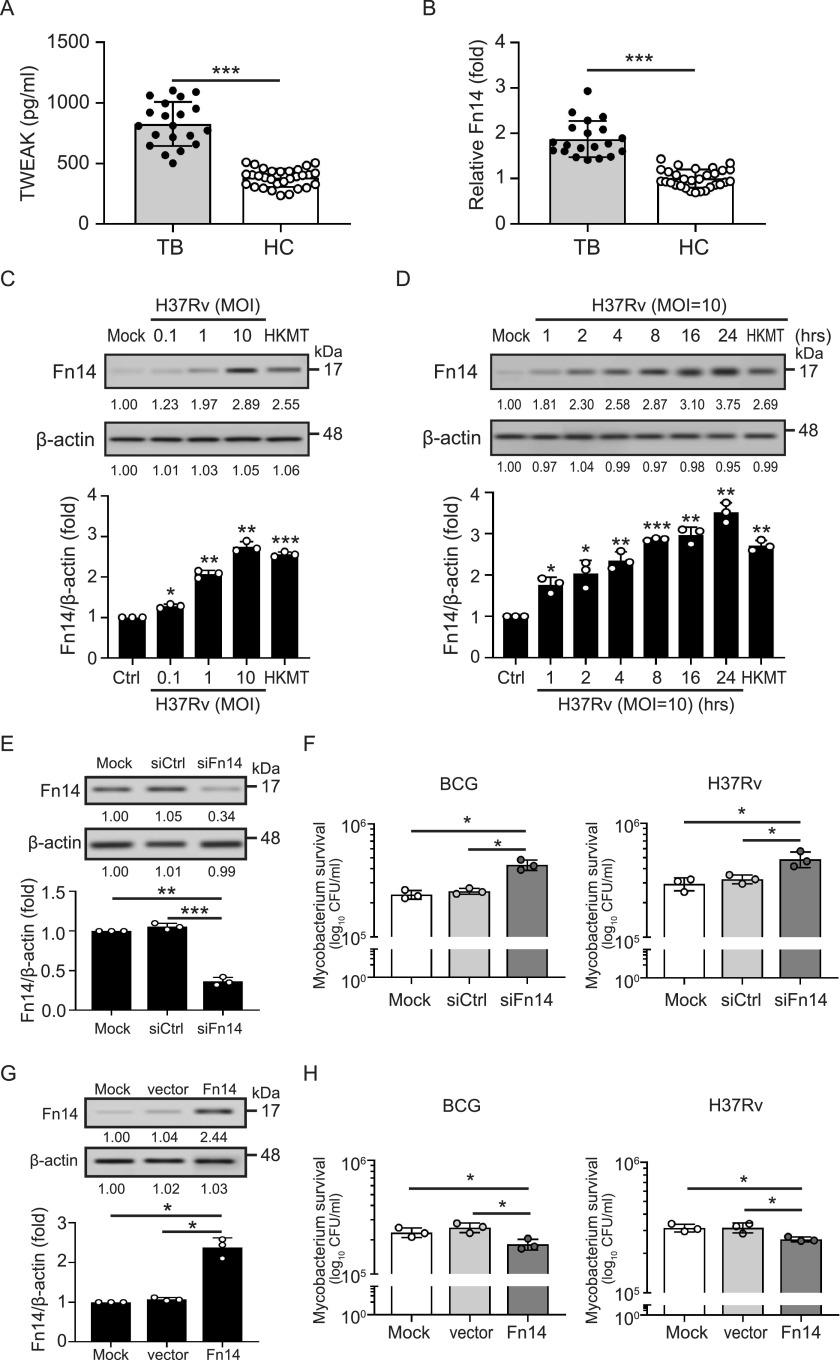
TWEAK-Fn14 signaling is involved in mycobacterial infection. Increased levels of (A) TWEAK secretion and (B) Fn14 expression in TB patients. (C) and (D) Fn14 expression is increased after mycobacterial infection in a (C) dose- and (D) time- dependent manner. (E-H) THP-1 cell-derived macrophages were transfected with (E) and (F) siFn14 to knock-down Fn14 or (F) and (G) pCMV-Fn14 to overexpress Fn14. After 48 h, cells were infected with M. bovis BCG or M. tuberculosis H37Rv at an MOI of 10 for 72 h. (E) and (G) The expression of Fn14 was detected using immunoblotting, and (F) and (H) mycobacterial survival was measured using CFU assay. Immunoblotting bands from β-actin were densitometrical measured by ImageJ to determine the lane normalization factor for samples. All experiments were performed in triplicate, and data are presented as the mean ± SD. An unpaired, two-tailed Student’s t-test was performed for between-group comparisons using GraphPad Prism software version 8. The image shown is from a single experiment that is representative of at least three separate experiments. *, *P < *0.05; **, *P < *0.01; ***, *P < *0.005.

Our *in vitro* results showed that Mycobacterium induced TWEAK upregulation (12). In order to validate this data, we analyzed the expression of TWEAK in patients with active TB. An increased level of TWEAK was revealed in the serum of active TB patients (*n* = 20, 826.5 ± 182.3 pg/mL, *P < *0.005) (Fig. 1A), compared to those in healthy controls (*n* = 30, 382.2 ± 75.7 pg/mL). We further analyzed the expression of Fn14 in the PBMCs of TB patients using qRT-PCR. The trend of Fn14 expression was similar to that of TWEAK, with elevated levels of Fn14 being observed in TB patients compared to those in healthy controls (1.87 ± 0.40-fold versus 1.00 ± 0.20-fold, *P < *0.005) (Fig. 1B). Moreover, Fn14 expression increased after M. tuberculosis H37Rv/BCG infection, or heat-killed M. tuberculosis (HKMT) treatment in a dose- (H37Rv [Fig. 1C], BCG [Fig. S1A], HKMT [Fig. S1C]) and time-dependent manner (H37Rv [Fig. 1D], BCG [Fig. S1B], HKMT [Fig. S1D]), respectively. The cell viability of THP-1 cell-derived macrophages was decreased after H37Rv infection, compared to those without infection (Fig. S1E) (72 h postinfection: 2.15 ± 0.07 × 10^4^ RLU versus 2.87 ± 0.05 × 10^4^ RLU, *P < *0.005). A similar trend was observed in cells with BCG infection (1.73 ± 0.03 × 10^4^ RLU versus 2.87 ± 0.05 × 10^4^ RLU, *P < *0.005).

### Fn14 inhibits intracellular mycobacterial growth.

To determine the biological function of Fn14 in mycobacterial infection, THP-1 cell-derived macrophages were transfected with siFn14 to knockdown Fn14 expression, and then the cells were infected with H37Rv at a multiplicity of infection (MOI) of 10. The dynamic of H37Rv growth in macrophages was measured using colony formation unit (CFU) assay (Fig. S1F). Increased mycobacterial survival was detected in Fn14 knockdown cells at 72h postinfection, compared to those in transfection control cells or mock cells (4.48 ± 0.24 × 10^5^ CFU/mL versus 3.24 ± 0.29 × 10^5^ CFU/mL versus 3.18 ± 0.41 × 10^5^ CFU/mL, *P < *0.05). To further confirm our observation, Fn14 knockdown cells or control cells were infected with BCG or H37Rv at an MOI of 10 for 72 h, respectively. The results showed that when intracellular Fn14 was knocked down efficiently (Fig. 1E), a higher survival of Mycobacterium was detected in Fn14-knockdown cells compared with control knockdown cells (BCG: 4.33 ± 0.45 × 10^5^ CFU/mL versus 2.53 ± 0.15 × 10^5^ CFU/mL, *P < *0.05; H37Rv: 4.84 ± 0.76 × 10^5^ CFU/mL versus 3.24 ± 0.29 × 10^5^ CFU/mL, *P < *0.05) (Fig. 1F). Conversely, when Fn14 was overexpressed (Fig. 1G), Mycobacterium survival was reduced (BCG: 1.83 ± 0.20 × 10^5^ CFU/mL versus 2.57 ± 0.25 × 10^5^ CFU/mL, *P < *0.05; H37Rv: 2.57 ± 0.10 × 10^5^ CFU/mL versus 3.15 ± 0.27 × 10^5^ CFU/mL, *P < *0.05) (Fig. 1H).

### TWEAK-induced autophagy through its receptor Fn14.

**FIG 2:**
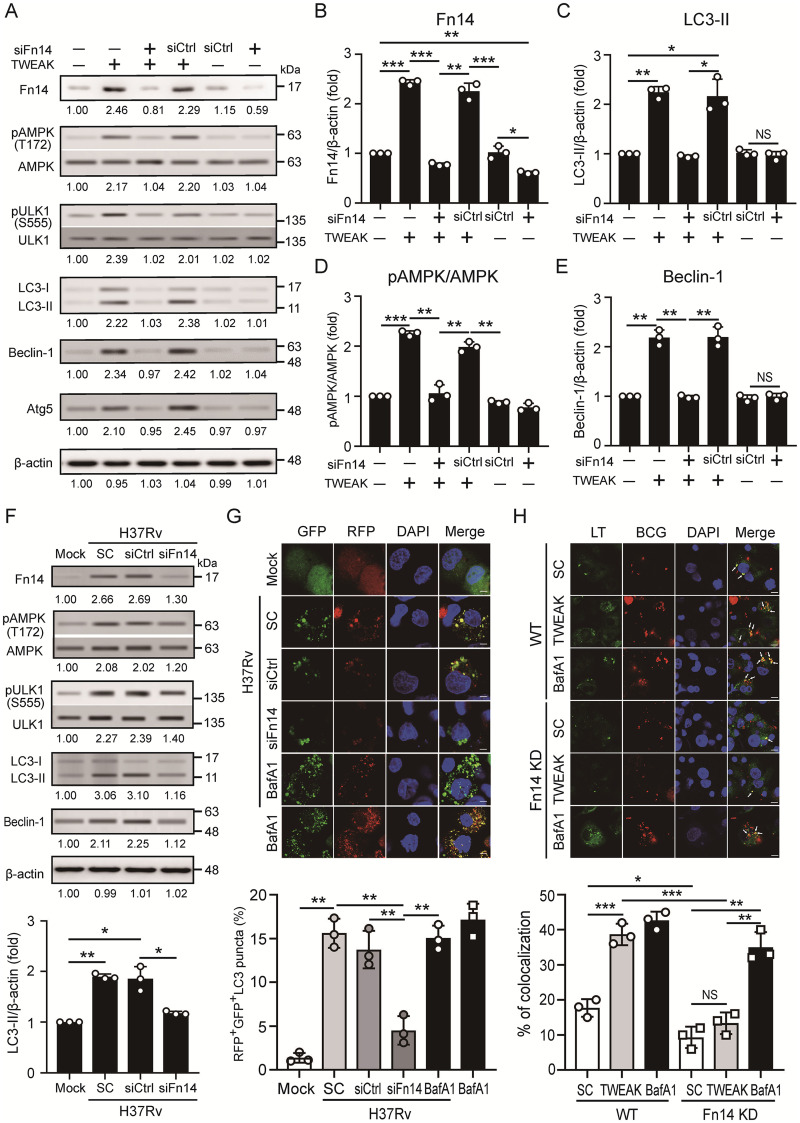
TWEAK-induced autophagy and mycobacterial phagosome maturation through Fn14. (A) to (E) THP-1 cell-derived macrophages were transfected with siFn14 to knockdown Fn14 expression, then cells were treated with TWEAK (100 ng/mL). The Fn14, AMPK phosphorylated at Thr172 (pAMPK), ULK1 phosphorylated at Ser555 (pULK1), Beclin-1, Atg5, LC3, and β-actin levels were detected and quantified using immunoblotting analysis. (F) Fn14 knockdown cells were infected with M. tuberculosis H37Rv at an MOI of 1 for 24 h. The levels of Fn14, phosphorylated AMPK, phosphorylated ULK1, Beclin-1, LC3, and β-actin were detected by immunoblotting. (G) THP-1 cells stably expressing RFP-GFP-LC3 fusion protein were transfected with Fn14 siRNA to knockdown Fn14. After 24 h, cells were infected with M. tuberculosis H37Rv at an MOI of 1 for 24 h. The RFP-GFP-LC3 puncta were detected by confocal microscopy (upper panel) and quantified (lower panel). Bafilomycin A1 (BafA1, 100 nM) treatment were used as positive control. (H) THP-1 cell-derived macrophages were treated with the indicated reagent for 24 h, then infected with Texas Red-labeled M. bovis BCG for 2 h and stained with LysoTracker Green (LT) dye. LT (green) and BCG (red) were detected by confocal microscopy (upper panel). The fraction (%) of mycobacterium-containing phagosomes colocalizing with LT was quantified (lower panel). RPMI medium were used as solvent control (SC). Immunoblotting bands from β-actin were densitometrical measured by ImageJ to determine the lane normalization factor for samples. All experiments were performed in triplicate, and data are presented as the mean ± SD. An unpaired, two-tailed Student’s t-test was performed for between-group comparisons using GraphPad Prism software version 8. The image shown is from a single experiment that is representative of at least three separate experiments. *, *P < *0.05; **, *P < *0.01; ***, *P < *0.005.

To determine whether TWEAK induces autophagy through its receptor Fn14, THP-1 cell-derived macrophages were transfected with siFn14 to knockdown Fn14 expression, and then the cells were treated with TWEAK (100 ng/mL). Significantly lower Fn14 expression was observed in cells transfected with siFn14 compared to control knockdown cells, indicating the efficiency of knock-down (0.61 ± 0.03-fold, *P < *0.005) (Fig. 2A and B). Increased levels of Fn14 were induced in naive (2.43 ± 0.06-fold, *P < *0.005) and control knockdown cells (2.26 ± 0.16-fold, *P < *0.005) after treating with TWEAK, but this effect was lacking in Fn14 knockdown cells (0.78 ± 0.03-fold, *P < *0.005). The results suggested that TWEAK induces Fn14 expression. Higher expression of LC3-II (Fig. 2A and C) was detected in naive cells (2.24 ± 0.11-fold, *P < *0.01) and control knockdown cells (2.16 ± 0.35-fold, *P < *0.05) 24 h post-TWEAK treatment; however, there was no significant difference in LC3-II expression induced in Fn14-knockdown cells with TWEAK stimulation (0.95 ± 0.03-fold). Similar trends were detected in phosphorylated AMPK (Fig. 2D) and Beclin-1 expression (Fig. 2E), suggesting that TWEAK-induced autophagy is Fn14-dependent.

We further investigated whether Fn14 could induce autophagy directly in the absence of TWEAK. As shown in Fig. S2, there was no significant difference in the expression of autophagy-related proteins (e.g., LC3-II, and Beclin-1) between Fn14 over-expressing cells and control cells in the absence of TWEAK, suggesting that Fn14 cannot induce autophagy directly and is required to interact with TWEAK to induce autophagy. In addition, we observed that there was no significant difference in cell viability between Fn14 over-expressing cells and control cells (2.81 ± 0.13 × 10^4^ RLU versus 2.88 ± 0.06 × 10^4^ RLU) (Fig. S1G). After H37Rv infection, the levels of decreased cell viability in Fn14 over-expressing cells was slightly more than control cells (1.98 ± 0.24 × 10^4^ RLU versus 2.15 ± 0.07 × 10^4^ RLU).

### TWEAK promotes mycobacterial phagosome maturation via Fn14.

Next, we investigated the role of Fn14 in mycobacterial phagosome maturation using Fn14 knockdown cells with H37Rv or BCG infection. Approximately 3-fold greater expression of LC3-II was detected in THP-1 cells 24 h post-H37Rv infection (*P < *0.005) (Fig. 2F); however, lower LC3-II expression was noted in Fn14-knockdown cells infected with H37Rv (*P < *0.005) compared with that in control knockdown cells or naive (RPMI medium treatment control, solvent control [SC]) cells. The effect of Fn14 on autophagic activity was further examined using THP-1 cells stably expressing the RFP-GFP-LC3 fusion protein. As shown in Fig. 2G, H37Rv infection induced autophagic flux was diminished when intracellular Fn14 was knocked down. The similar results were shown in cells with BCG infection (Fig. S3). Taken together, our data suggested that Fn14 plays a key role in Mycobacterium-induced autophagy.

To confirm the role of TWEAK-Fn14 in mycobacterial phagosome maturation, wild type or Fn14 knockdown cells were infected with Texas Red-labeled BCG at an MOI of 10, and the formation of autophagosomes containing BCG was examined using confocal microscopy. Our results showed elevated autophagosome maturation, reflected by co-localization of BCG with LysoTracker dye in TWEAK-treated cells compared with control cells (38.7 ± 3.1 versus 17.7 ± 2.5%, *P < *0.005) (Fig. 2H). Reduced autophagosome maturation was revealed in Fn14-knockdown cells with BCG infection, compared to that in THP-1 naive cells (9.3 ± 3.1 versus 17.7 ± 2.5%, *P < *0.05), suggesting that Fn14 plays a crucial role in autophagosome maturation. In addition, the effect of TWEAK on the induction of mycobacterial autophagosome formation was not significant in Fn14 knockdown cells (13.3 ± 3.1 versus 9.3 ± 3.1%). These findings suggest that Fn14 is required for TWEAK-promoted mycobacterial autophagosome formation and maturation.

### TWEAK-Fn14 induced autophagy is reactive oxygen species-dependent.

**FIG 3:**
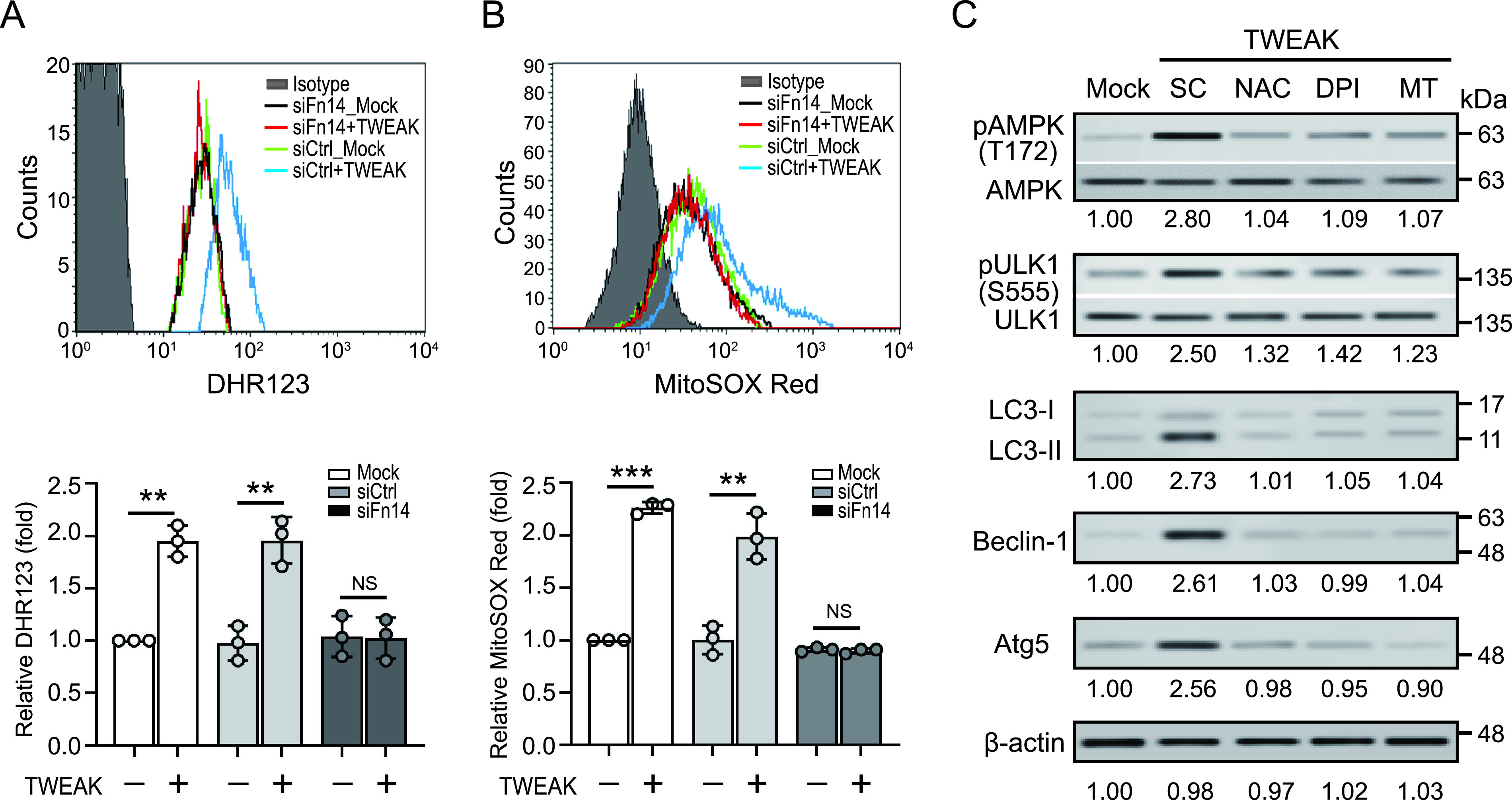
TWEAK-Fn14-induced autophagy is ROS-dependent. TWEAK-induced elevated (A) cytosolic ROS, and (B) mitochondrial ROS production in THP-1 cells is Fn14-dependent (C). THP-1 cell-derived macrophages were treated with TWEAK (100 ng/mL) in the absence or presence of the ROS scavenger N-acetyl-L-cysteine (NAC), the NOX inhibitor diphenyleneiodonium (DPI), or the mitochondria-specific superoxide scavenger MitoTEMPO (MT) for 24 h. AMPK phosphorylated at Thr172 (pAMPK), total AMPK, ULK1 phosphorylated at Ser555 (pULK1), total ULK1, Beclin-1, Atg5, LC3, and β-actin levels were detected by an immunoblotting analysis. PBS was used as solvent control (SC). Immunoblotting bands from β-actin were densitometrical measured by ImageJ to determine the lane normalization factor for samples. All experiments were performed in triplicate, and data are presented as the mean ± SD. An unpaired, two-tailed Student’s t-test was performed for between-group comparisons using GraphPad Prism software version 8. The image shown is from a single experiment that is representative of at least three separate experiments. *, *P < *0.05; **, *P < *0.01; ***, *P < *0.005.

Accumulating evidence has shown that autophagy is regulated by reactive oxygen species (ROS) (16). We observed that TWEAK induces the production of cytosolic ROS (1.95 ± 0.15-fold, *P < *0.01) (Fig. 3A) and mitochondrial ROS (mROS) (2.26 ± 0.06-fold, *P < *0.005) (Fig. 3B), which is consistent with other reports (17, 18). In addition, we showed that the effects of TWEAK on the production of cytosolic ROS and mROS were both diminished when Fn14 was knocked down, suggesting that TWEAK promotes cytosolic ROS/mROS production by interacting with Fn14.

We further investigated whether TWEAK-Fn14 induces autophagy by regulating ROS production. Nicotinamide-adenine dinucleotide phosphate oxidase (NOX) is crucial for the production of ROS. As shown in Fig. 3C, TWEAK-induced activation of AMPK, and the expression of autophagy-related proteins LC3-II, Beclin-1, and Atg5, were almost completely inhibited in the presence of the ROS scavenger N-acetyl-L-cysteine (NAC), the NOX inhibitors diphenyleneiodonium (DPI), and the mitochondria-specific superoxide scavenger MitoTEMPO (MT). Our results revealed that ROS play a crucial role in TWEAK-Fn14-induced autophagy.

### TWEAK-Fn14 induced autophagy by promoting calcium influx.

**FIG 4:**
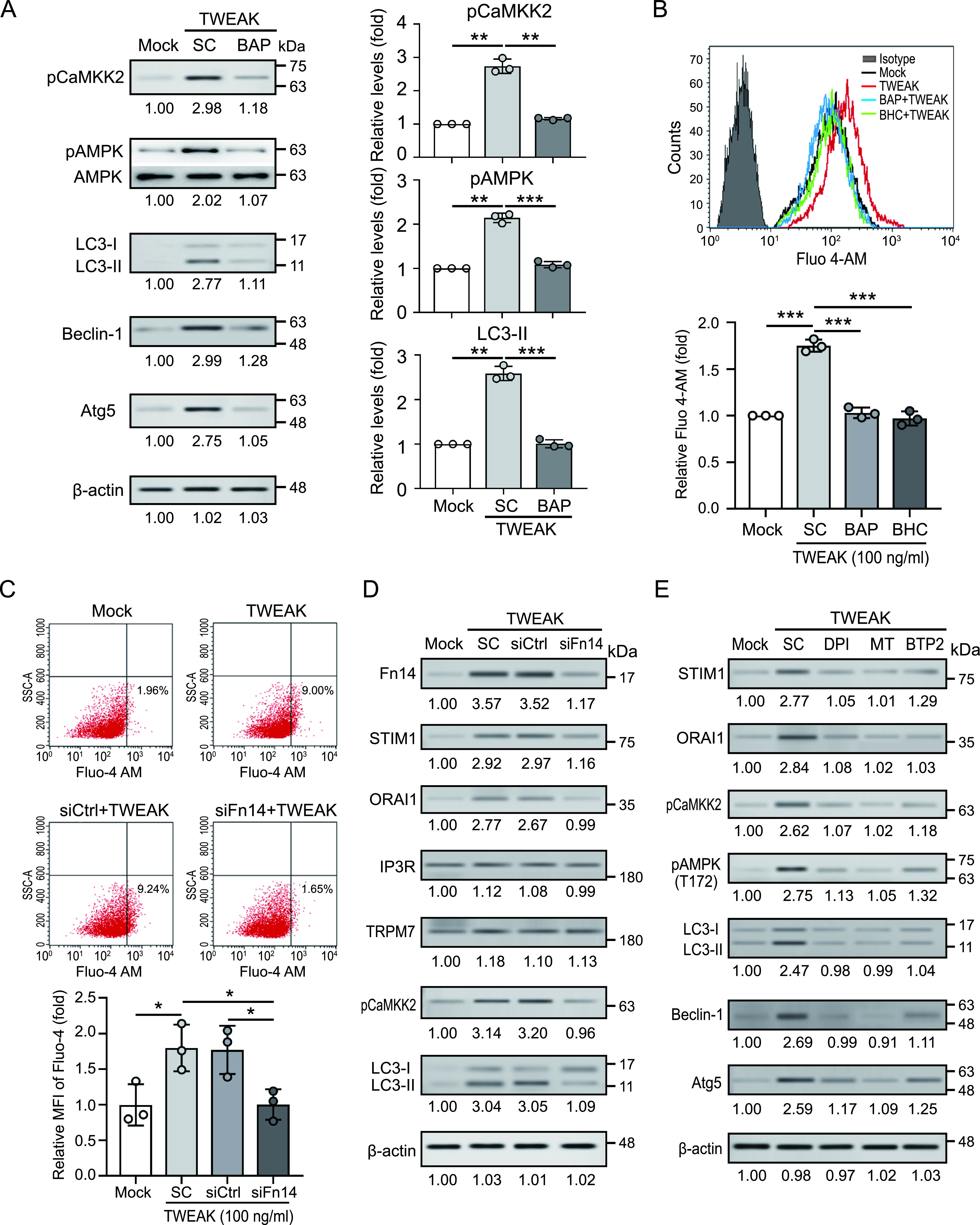
TWEAK-Fn14 axis-induced oxidative stress promotes calcium-associated autophagy by activating STIM1-ORAI1 calcium channel. (A) THP-1 cell-derived macrophages were stimulated with TWEAK (100 ng/mL) in the presence of the intracellular calcium chelator BAPTA-AM (10 μM). Phosphorylated CaMKK2, AMPK phosphorylated at Thr172 (pAMPK), autophagy-associated proteins, and β-actin levels were detected and quantified using immunoblotting analysis. (B) THP-1 cell-derived macrophages were stained with Fluo-4 AM (5 μM, 1h at 37°C), then treated with TWEAK for 10 min in the presence of BAPTA-AM or bepridil hydrochloride. Calcium mobilization in Fluo-4 AM loading cells was detected and quantified using flow cytometry. (C) Fn14 knockdown or control cells were stained with Fluo-4 AM, then treated with TWEAK (100 ng/mL) for 10 min. The calcium mobilization in Fluo-4 AM loading cells was detected and quantified using flow cytometry. (D) Fn14 knockdown or control cells were treated with TWEAK for 24 h. The Fn14, calcium channel, and autophagy-associated proteins were detected using immunoblotting analysis. (E) THP-1 cell-derived macrophages were treated with TWEAK in the absence or presence of the NOX inhibitor diphenyleneiodonium (DPI), the mitochondria-specific superoxide scavenger MitoTEMPO (MT), or the Ca^2+^ release-activated Ca^2+^ (CRAC) channel inhibitor BTP2 for 24 h. The STIM1, ORAI1, and autophagy-associated proteins were detected using immunoblotting analysis. PBS was used as solvent control (SC). Immunoblotting bands from β-actin were densitometrical measured by ImageJ to determine the lane normalization factor for samples. All experiments were performed in triplicate, and data are presented as the mean ± SD. An unpaired, two-tailed Student’s t-test was performed for between-group comparisons using GraphPad Prism software version 8. The image shown is from a single experiment that is representative of at least three separate experiments. *, *P < *0.05; **, *P < *0.01.

Chinopoulos et al. have revealed that there is a close association between oxidative stress and calcium influx (19). We demonstrated that TWEAK-Fn14 induced autophagy through AMPK activation (12), and Hurley et al. (20) found that AMPK could be activated by calcium/calmodulin-dependent protein kinase kinase 2 (CaMKK2), which is controlled by an increase in intracellular calcium ions. Therefore, we investigated whether TWEAK-Fn14 induced autophagy by regulating calcium influx. The immunoblotting results showed that TWEAK-Fn14 induced CaMKK2 activation and increased autophagy-related protein expression (Fig. 4A). This effect was suppressed in the presence of the Ca^2+^ chelator BAPTA-AM. This result suggests that TWEAK-Fn14-mediated autophagy is associated with calcium ions. We further assessed the association between TWEAK-Fn14 signaling and the variation in cytosolic calcium concentration using Fluo-4 AM calcium indicator dye combined with flow cytometry. Increased intracellular calcium levels were detected in cells treated with TWEAK (1.75 ± 0.07-fold, *P < *0.005) (Fig. 4B), while calcium concentration was inhibited in the presence of BAPTA-AM or bepridil hydrochloride (BHC, a calcium channel modulator). Moreover, the effect of TWEAK on the intracellular calcium influx was diminished when Fn14 was knocked down (Fig. 4C), suggesting that TWEAK enhances calcium influx by interacting with Fn14.

### TWEAK-Fn14 enhances STIM1-ORAI1 expression by promoting ROS production.

We further analyzed the variation in calcium channel expression under TWEAK-Fn14 axis activation (Fig. 4D). The results showed that elevated levels of calcium channel ORAI1 and its activator STIM1 were detected in THP-1-derived macrophages after TWEAK treatment, but this effect was lacking when Fn14 was deficient. Additionally, there was no significant difference in expression of other calcium channels, including IP3R (IP3 [inositol 1,4,5-trisphosphate] receptor), and TRPM7 (transient receptor potential cation channel subfamily M member 7). We further confirmed the association between TWEAK-Fn14 signaling-induced oxidative stress and calcium-autophagy using pharmacological inhibition. As shown in Fig. 4E, TWEAK-Fn14 stimulated STIM1-ORAI1 upregulation, CaMKK2 activation, and inducing autophagy-associated proteins expression. But this effect was diminished in the presence of ROS inhibitor (DPI or MitoTEMPO) and calcium release-activated channels (CRAC) inhibitor (BTP2). Taken together, our results show that the TWEAK-Fn14 axis upregulates STIM1-ORAI1 expression by promoting oxidative stress, thus causing a calcium influx that activates CaMKK2 and autophagy-related proteins.

### TWEAK-Fn14 enhances calcium-associated autophagy against mycobacterial infection.

**FIG 5:**
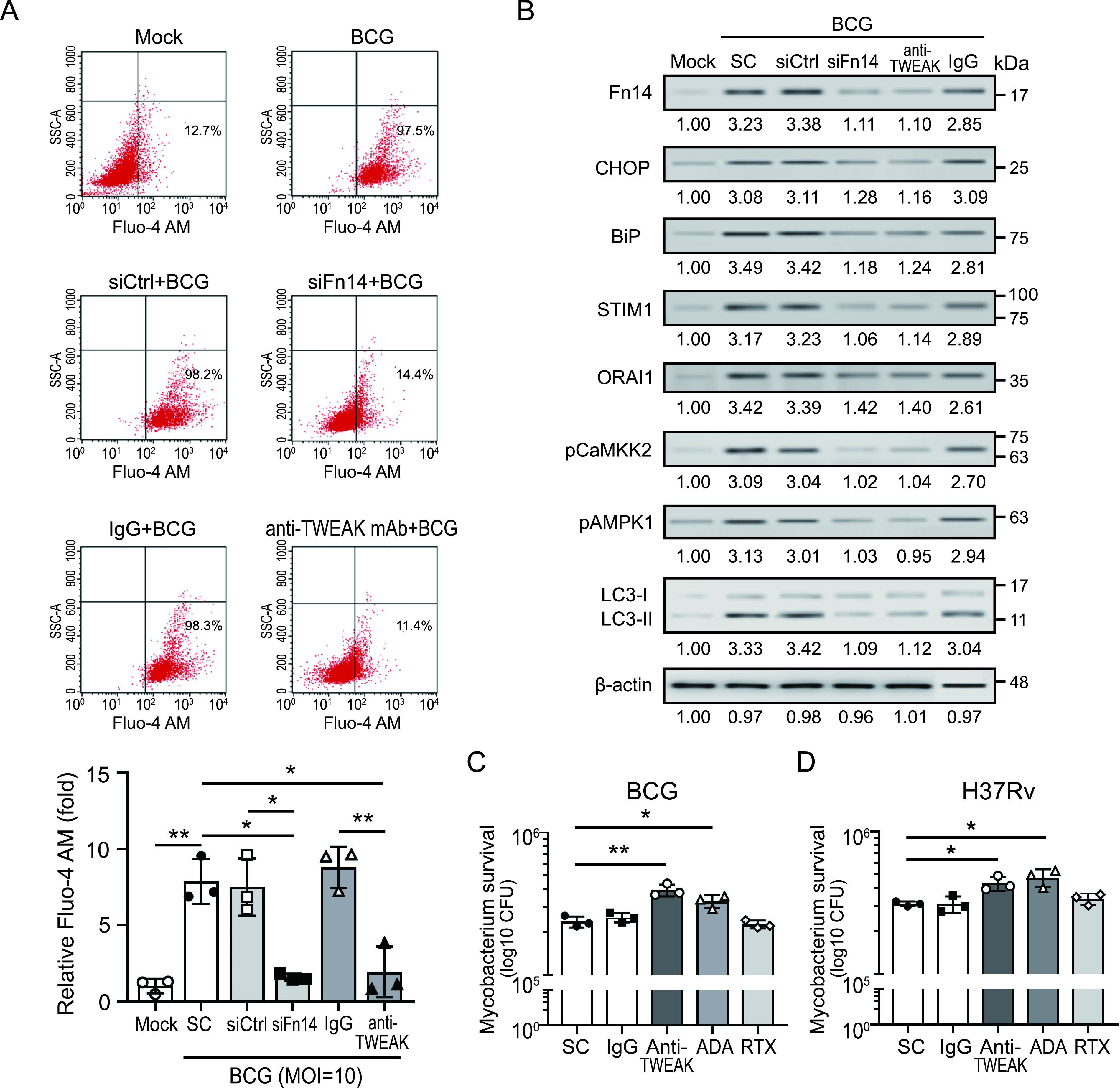
TWEAK-Fn14 enhances calcium-associated autophagy against mycobacterial infection. THP-1 cell-derived macrophages in the absence or presence of anti-TWEAK antibodies, or Fn14 knockdown cells were stained with Fluo-4 AM (5 μM, 1h at 37°C), then infected with M. bovis BCG at an MOI of 10 for 20 min. (A) Calcium mobilization in Fluo-4 AM loading cells was detected and quantified using flow cytometry. (B) The Fn14, ER stress markers, STIM1, ORAI1, and autophagy-associated proteins were detected using immunoblotting analysis. (C and D) THP-1 cell-derived macrophages were infected with (C) M. bovis BCG or (D) M. tuberculosis H37Rv at an MOI of 10 in the presence of individual biological (10 μg/mL) or anti-TWEAK antibodies (10 μg/mL). Mycobacterial survival was determined by a CFU assay at 72 h postinfection. RPMI medium were used as solvent control (SC). Immunoblotting bands from β-actin were densitometrical measured by ImageJ to determine the lane normalization factor for samples. All experiments were performed in triplicate, and data are presented as the mean±SD. An unpaired, two-tailed Student’s t-test was performed for between-group comparisons using GraphPad Prism software version 8. The image shown is from a single experiment that is representative of at least three separate experiments. *, *P < *0.05; **, *P < *0.01. ADA, adalimumab; RTX, rituximab.

Next, we investigated the role of TWEAK-Fn14 axis-induced calcium-associated autophagy in mycobacterial infection. We observed that mycobacterial infection induced increased levels of cytosolic calcium (Fig. 5A and Fig. S4), which is consistent with another report (21). However, this effect was diminished when intracellular Fn14 was knocked down or in the presence of TWEAK blocker, suggesting that the TWEAK-Fn14 axis plays a key role in mycobacteria-induced intracellular calcium upregulation. We further investigated the association between TWEAK-Fn14 signaling and calcium-associated autophagy in mycobacterial infection. As shown in Fig. 5B, STIM1 and ORAI1 were both increased in THP-1 cells with BCG infection, and were accompanied by upregulation of endoplasmic reticulum (ER) stress marker C/EBP Homologous Protein (CHOP) and BiP. However, these effects were suppressed in Fn14 knock-down cells or in the presence of anti-TWEAK blocker. Our results show that TWEAK-Fn14 signaling promotes calcium-associated autophagy against mycobacterial infection, maybe by promoting oxidative stress to induce ER stress, and activate STIM1-ORAI1 calcium channel to increase intracellular Ca^2+^ levels.

TWEAK has been considered as a potential target for the development of novel therapeutics. The biologic agent (BIIB023) that blocks TWEAK has completed a phase II clinical trial in rheumatic diseases therapy (22). We assessed the effects of TWEAK blocker on mycobacterial survival. Our results revealed that adalimumab (TNF-α-blocker) affects the host response, resulting in higher mycobacteria survival that is consistent with a previous report (23). In addition, the higher survival of mycobacteria was detected in cells treated with TWEAK blocker than in control cells (BCG: 3.94 ± 0.22 × 10^5^ CFU/mL versus 2.37 ± 0.21 × 10^5^ CFU/mL, *P < *0.01 [Fig. 5C]; H37Rv: 4.31 ± 0.51 × 10^5^ CFU/mL versus 3.10 ± 0.12 × 10^5^ CFU/mL, *P < *0.05, [Fig. 5D]). In conclusion, our results indicate that TWEAK-Fn14 blocker could inhibit calcium-associated autophagy in mycobacterial infection and thus enhance mycobacteria survival.

### TWEAK-Fn14 signaling induces cell death in late phase of BCG infection by inducing mitochondrial ROS production.

**FIG 6:**
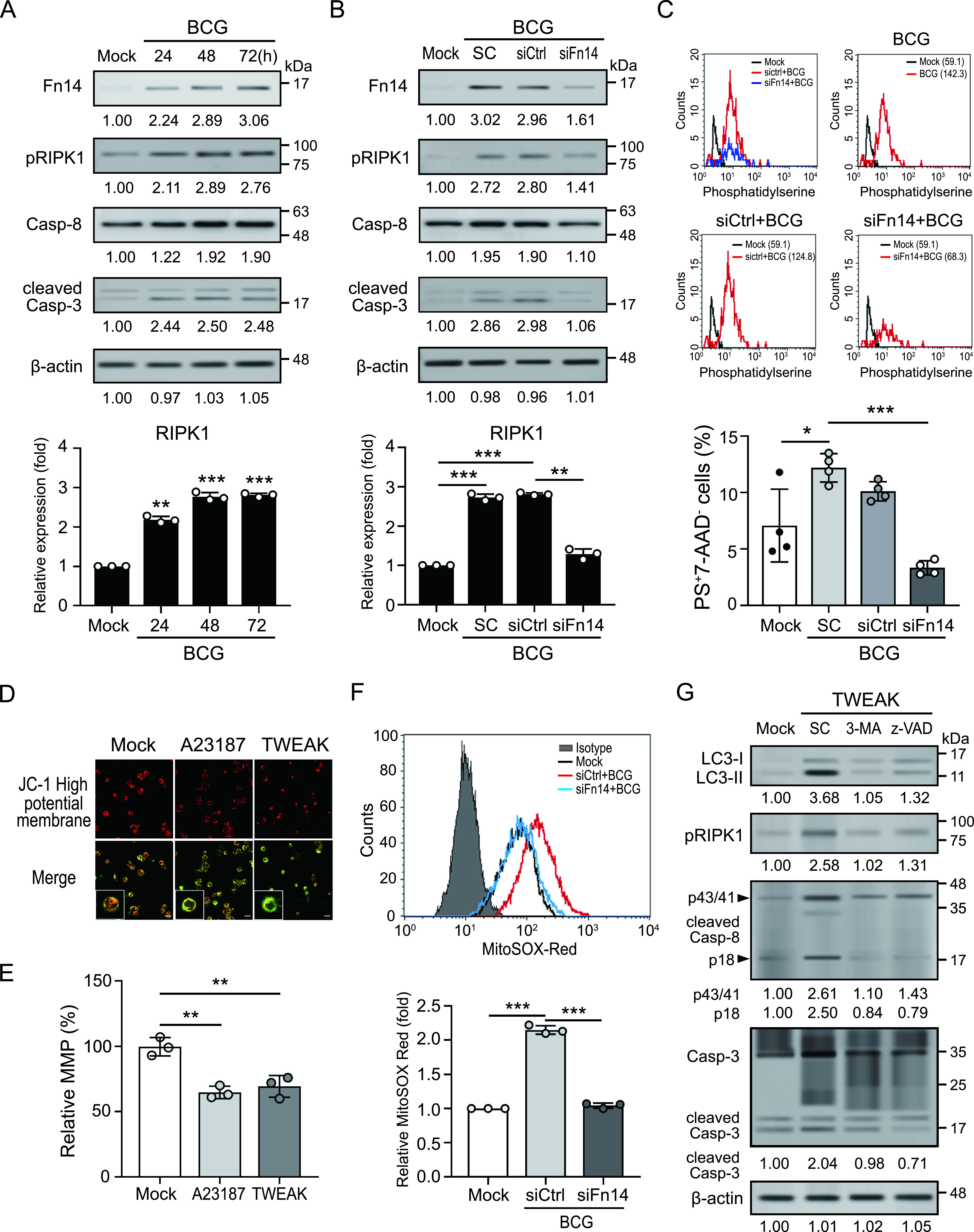
Increased TWEAK-Fn14 signaling induces cell death in late phase of mycobacterial infection by inducing mitochondrial ROS production. (A) Elevated Fn14 expression and increased levels of cell death-associated markers were found in THP-1-derived macrophages after BCG infection for 72 h, and (B) were diminished in Fn14 knockdown cells. (C) An increase in the percentage of phosphatidylserine^+^7-AAD- (early stage of apoptosis) cells were observed in cells infected with BCG, but this effect was decreased in Fn14 knockdown cells. (D) and (E) THP-1 -derived macrophages were treated with the calcium ionophore A23187, or TWEAK. (D) The morphology of mitochondria was observed using a confocal microscope with MITO-ID dye. (E) The mitochondrial membrane potential (MMP) was measured using a plate reader with JC-1 staining. (F) The levels of mitochondrial ROS production in THP-1-derived macrophages under different treatments were analyzed using flow cytometry with MitoSOX Red staining. (G) THP-1-derived macrophages were treated with TWEAK in the presence of 3-MA (5 mM) or apoptosis inhibitor z-VAD-FMK, (10 mM). The autophagy- and cell death-associated proteins were detected using immunoblotting. RPMI medium were used as solvent control (SC). Immunoblotting bands from β-actin were densitometrical measured by ImageJ to determine the lane normalization factor for samples. All experiments were performed in triplicate, and data are presented as the mean ± SD. An unpaired, two-tailed Student’s t-test was performed for between-group comparisons using GraphPad Prism software version 8. The image shown is from a single experiment that is representative of at least three separate experiments. *, *P < *0.05; **, *P < *0.01.

Autophagy can protect cells against death, but it can also mediate cellular demise, depending on the specific circumstances (24). Previous studies demonstrated that macrophages infected with attenuated Mycobacterium BCG become apoptotic, which limits bacterial replication and contributes antigen presentation (25). TWEAK-Fn14 signaling could induce multiple pathways of cell death (26), therefore, we assessed the effects of TWEAK-Fn14 on cell death during BCG infection. Elevated Fn14 expression accompanied by increased cell death-associated proteins (e.g., receptor-interacting protein kinase 1 [RIPK1], caspase 8, and cleavage caspase 3) were found in THP-1-derived macrophages after BCG infection for 72 h (Fig. 6A). However, this effect was suppressed in Fn14 knockdown cells (Fig. 6B), suggesting that TWEAK-Fn14 signaling is involved in the regulation of cell death during BCG infection. We further confirmed our result using an apoptosis/necrosis assay. An increased percentage of phosphatidylserine^+^7-AAD- (early stage of apoptosis) cells was revealed in naive cells or transfection control cells infected with BCG, compared to uninfected cells (Fig. 6C). But this effect was decreased in Fn14 knockdown cells. The mitochondrial membrane potential (MMP) plays a key role in early stages of apoptosis (27). Our results showed that TWEAK-Fn14 induced Ca^2+^ influx and accumulating evidence has revealed that Ca^2+^ influx affects MMP (28). Therefore, we analyzed the effect of TWEAK on MMP using MITO-ID dye (Fig. 6D) and tetraethyl benzimidazolyl carbocyanine iodide (JC-1) staining (Fig. 6E). The hypopolarized mitochondria lined the plasma membrane in cells treated with Ca^2+^ ionophore A23187 or TWEAK, in contrast to the evenly distributed hyperpolarized mitochondria in control cells (Fig. 6D). The MMP was decreased in cells after treatment with TWEAK (Fig. 6E). Additionally, elevated mROS were induced in cells after BCG infection compared to those without infection (Fig. 6F). But there was no significant difference in the Fn14 knockdown cell, suggesting that TWEAK-Fn14 signaling could induce mROS production and thus lead to cell death in late mycobacterial infection. Finally, we assessed the effect of TWEAK-Fn14 signaling induced autophagy on cell death using pharmacological inhibition. THP-1-derived macrophages were treated with TWEAK in the presence of an autophagy antagonist (3-methyladenine, 3-MA, 5 mM) or apoptosis inhibitor (N-Benzyloxycarbonyl-Val-Ala-Asp[O-Me] fluoromethyl ketone, z-VAD-FMK, 10 mM). The autophagy- and cell death-associated proteins were detected using immunoblotting (Fig. 6G). The results revealed that TWEAK-induced activation of RIPK1, and caspase-3 were both diminished in the presence of 3-MA, suggesting that TWEAK-Fn14 signaling may cause cell death in BCG infection by inducing autophagy.

## DISCUSSION

Tuberculosis remains an important infectious disease and cause of mortality worldwide. The recent appearance of multiple drug-resistant strains has resulted in an even greater threat. There is an urgent need to understand how M. tuberculosis survives in host macrophages so that alternative therapeutic strategies can be developed. Autophagy plays a crucial role in protecting the host against pathogens. In thisstudy, we found that TWEAK-Fn14 signaling promotes oxidative stress, thus inducing autophagy against mycobacterial infection by activating STIM1-ORAI1 calcium channels and inducing calcium influx.

In order to survive, mycobacteria develop a variety of immune evasion strategies against autophagy and to persist within macrophages. Romagnoli et al. demonstrated that M. tuberculosis H37Rv impairs autophagic flux and is ESAT-6 secretion system-1 (ESX-1)-dependent (29). The attenuated BCG strain was not able to hamper autophagosome maturation, however, which is caused by the deletion of the region of difference 1 (RD1) locus, resulting in the loss of ESX-1 (30). We demonstrated previously that mycobacteria (BCG/H37Rv), and heat-killed M. tuberculosis (HKMT) could induce TWEAK expression (12). In this study, we further showed that both mycobacterial infection (BCG/H37Rv), and HKMT treatment could induce Fn14 upregulation, suggesting that the mycobacterial components contribute to TWEAK-Fn14 upregulation in THP-1 cells. We demonstrated that TWEAK-Fn14 axis promotes mycobacterial phagosome maturation via enhancing ROS production. Although we showed that TWEAK-Fn14 axis suppressed mycobacterial growth, the inhibitory effect on BCG strain seems stronger than H37Rv. Therefore, the association between the inhibitory effect of TWEAK-Fn14 axis and ESX-1/RD1 on mycobacterial survival requires further in-depth experiments.

AMPK is activated by phosphorylation of the α subunit, which is catalyzed by CaMKK2 (29). In this study, we found that TWEAK increases the levels of intracellular calcium by interacting with Fn14 to induce autophagy by activating CaMKK2. Calcium functions as a second messenger that is important in regulating multiple fundamental physiological functions. Accumulating evidence has revealed that intracellular calcium plays a key role in the regulation of autophagy and phagosomal maturation (31–33). Francis et al. (34) demonstrated that M. tuberculosis ESAT-6 induces calcium influx to contribute to the necrotic pathology necessary for granuloma formation. Additionally, mycobacteria interact with the actin binding protein coronin 1 to activate the phosphatase calcineurin, thereby preventing phagosomal maturation (35). However, Malik et al. (36) suggested that mycobacteria inhibit calcium signaling to reduce phagosome–lysosome fusion and increase survival within human macrophages. Recently, miR-27a has been demonstrated to control the intracellular survival of M. tuberculosis by regulating calcium-associated autophagy (37). In this study, we showed that TWEAK-Fn14 signaling regulates calcium influx to promote phagosomal maturation and, thus, control mycobacterial survival. When cellular Fn14 is deficient or in the presence of the anti-TWEAK antibody, the levels of mycobacteria-induced calcium influx were decreased, resulting in activation of CaMKK2 and suppressing the expression of autophagy-related proteins, thus increasing mycobacterial survival.

Autophagy is a response to diverse cellular stressors, which are closely associated with oxidative stress (16). Xenophagy (canonical autophagy) and LC3-associated phagocytosis (LAP, non-canonical autophagy) are different autophagy mechanism against mycobacterial infection (38), but ROS production is essential in both xenophagy (39) and LAP (40). Madrigal-Matute et al. demonstrated that TWEAK-Fn14 interaction promotes oxidative stress through NADPH oxidase activation in macrophages (17). We showed that the TWEAK-Fn14 axis increases ROS production, followed by calcium influx promotion and CaMCCK2-associated autophagy activation, thus inhibiting mycobacteria survival. When ROS production was inhibited, the TWEAK-Fn14 axis-induced autophagy was suppressed. Our results suggest that the TWEAK-Fn14 axis may regulate both mechanisms of autophagy (xenophagy and LAP) to control mycobacterial infection by promoting ROS production. Further in-depth experiments are required to validate our hypothesis.

In addition to NOX-2-associated ROS, we observed that TWEAK-Fn14 signaling promoted mROS production, which is consistent with other reports (18, 41). A previous study demonstrated that TWEAK induces mitochondrial oxidative stress, accompanied by the release of proinflammatory cytokines (41). Roca et al. demonstrated that TNF mediates the host response to mycobacteria via mROS production (42). We showed that TWEAK-Fn14 signaling induced oxidative stress and increased ER stress during mycobacterial infection. Elevated ER stress may cause impaired calcium and redox homeostasis and oxidative stress via protein overload, thereby also influencing vital mitochondrial functions (43). Increased cytosolic Ca^2+^ and increased Ca^2+^ influx in mitochondria from ER can stimulate mitochondria to produce more mROS (44). More in-depth studies are required to determine the detailed mechanism of TWEAK-Fn14 signaling in mROS production.

Accumulating evidence has demonstrated that redox modifications could regulate Ca^2+^ channel expression (45). Store-operated calcium entry (SOCE) is a major mechanism for Ca^2+^ regulation that is mediated by Ca^2+^ release-activated Ca^2+^ (CRAC) channels (46). CRAC channels are hexamers of ORAI1 proteins located in the plasma membrane that are activated by STIM1 located in the ER. STIM1 and ORAI1 constitute the core machinery of the ubiquitous SOCE pathway and loss of function in these proteins is associated with severe immune and muscular disorders and infectious diseases (47). Kahlfuss et al. demonstrated that STIM1-mediated calcium influx controls anti-fungal immunity (48). Dramatically increased STIM1 was observed in sera of patients with influenza A virus infection, and this mediated virus-induced oxidative stress and inflammation (49). Desvignes et al. found that STIM1 controls multiple aspects of T cell–mediated immune regulation to limit injurious inflammation during chronic M. tuberculosis infection (50). In addition to being a sensor for intracellular Ca^2+^ stores, Hawkins et al. demonstrated that STIM1 functions as a redox sensor to constitutively activate CRAC channels under oxidative conditions (51). Herein, we observed that TWEAK-Fn14-induced calcium-associated autophagy was accompanied by STIM1-ORAI1 upregulation. However, this effect was suppressed in the presence of ROS inhibitor, suggesting that TWEAK-Fn14 induced calcium-associated autophagy by promoting oxidative stress to activate the STIM1-ORAI1 axis. Our results also showed that if STIM1-ORAI1 is blocked using a CRAC channel inhibitor, TWEAK-induced autophagy will be suppressed. But the mediator between TWEAK-Fn14 induced oxidative stress and STIM1-ORAI1 remains unknown, and more in-depth studies are needed to confirm the mechanism.

Fn14 is expressed at low levels in normal tissues but is highly expressed following tissue injury (52). The TWEAK-Fn14 axis is multi-functional and involved in the pathogenesis of many diseases, including autoimmune diseases, cardiovascular disease, and nephritic disease (13, 53, 54). Persistent activation of the TWEAK-Fn14 pathway may contribute to the pathology of these diseases. Blockade or deficiency of Fn14 (or TWEAK) had favorable therapeutic effects in a variety of disease models, and thus it has been considered as a potential therapeutic target (15, 55). We showed that the TWEAK-Fn14 axis plays a novel role in anti-mycobacterial infection by regulating calcium-associated autophagy. Mycobacterial survival increased when cells were Fn14-deficient or under anti-TWEAK treatment. Larger studies are needed to confirm the effects of TWEAK inhibitor and Fn14 deficiency on latent TB reactivation.

Ca^2+^ and ROS are key factors of cell death (45). Accumulating evidence has shown that M. tuberculosis exerts finely balanced control over the mode of death of infected host cells (56). Excess TNF-α triggers programmed necrosis of infected macrophages by inducing mROS, which activates the cytosolic protein BAX, thus promoting calcium flow from the ER into the mitochondrion, and the resultant mitochondrial calcium overload triggers cyclophilin-d-mediated necrosis (57). A previous study observed that TWEAK induces TNF-α and drives TNFR1-dependent apoptosis signaling (8). Martin-Sanchez et al. reported that TWEAK-Fn14 and RIPK1 mediate cell death during acute kidney injury (58). But the role of TWEAK in cell death during mycobacterial infection remains unclear. O'Sullivan et al. demonstrated that both the mitochondria and the lysosomes play key roles in caspase-independent cell death in macrophages after M. tuberculosis infection (59). Herein, we showed that TWEAK-Fn14 signaling induced ER stress, mROS production, and autophagy against mycobacterial infection by promoting oxidative stress and modulating calcium influx. Moreover, we observed that mycobacterial infection induced TWEAK-Fn14 signaling in a time-dependent manner. Persistently TWEAK-Fn14 signaling reduced mitochondrial membrane potential and caused mitochondrial ROS accumulation, thus activating cell death-associated proteins in late mycobacterial infection. On the other hand, Ikner et al. demonstrated that TWEAK triggers apoptosis by promoting assembly of a RIPK1-FADD (Fas-associated death domain)-caspse-8 complex to induce caspase-8 activation (8). Our results revealed that TWEAK-Fn14-induced autophagy is associated with capsase-8 expression and its activation. We speculate that persistently TWEAK-Fn14 signaling could induce activation of RIPK1 and caspase-8, thus leading apoptosis to against mycobacterial infection. Further in-depth *ex-vivo* studies are required to confirm the effects of the TWEAK-Fn14 axis on cell death in tuberculosis.

We observed previously that there is no significant difference of mycobacterial growth in Fn14-overexpressing Raw264.7 cells, compared to those in naive Raw264.7 cell or vector transfection cells. Polek et al. (60) demonstrated that Raw264.7 cells do not express Fn14, and they proposed that TWEAK mediated signal transduction by binding to 1 of at least 2 distinct receptors. Combining the published data and our result, we speculate that the effects on TWEAK-Fn14 axis on mycobacterial infection may be specific in human macrophages. Further in-depth experiments are required to validate our hypothesis. Because of the limitations of biosafety criteria and space in our institute means that we were not able to perform animal experiments to validate our results. We validated our observations using an *in vitro* cell-based assay and clinical specimens from TB patients, suggesting that our results still provide valuable information.

**FIG 7:**
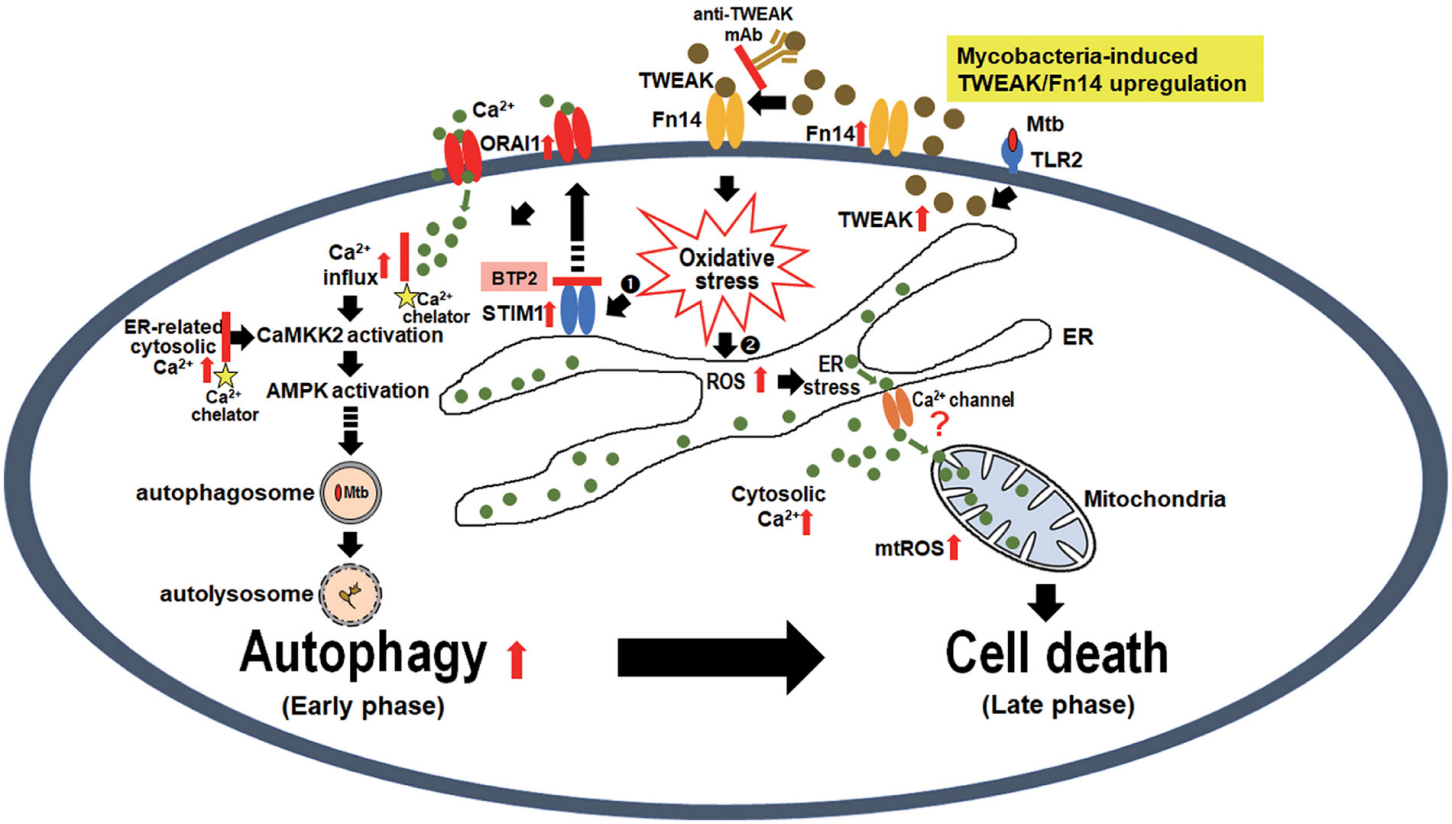
Proposed model for the regulatory mechanism of TWEAK-Fn14 signaling in autophagy and cell death during mycobacterial infection based on the results of this study. TWEAK-Fn14 signaling promotes oxidative stress to enhance STIM1 expression, and its activation of the Ca^2+^ channel ORAI1. Elevated calcium influx stimulated the activation of CaMCCK2 and its downstream effector AMPK, thus inducing autophagy in the early phase of infection. Persistently TWEAK-Fn14 signaling caused cell death in macrophages in the late phase of infection by reducing mitochondrial membrane potential–caused mitochondrial ROS accumulation, and activating cell death-associated proteins. Genetic Fn14 deficiency, TWEAK blocker or calcium chelator treatment decreased the oxidative stress-induced calcium influx, thus suppressing autophagy and cell death in mycobacteria-infected macrophages, resulting in elevated mycobacterial survival.

Mycobacterial infection represents a dynamic balance between the host and pathogen. TNF-α plays a key role in promoting the activation of innate cells and the release of pro-inflammatory cytokines. However, TWEAK balances TNF-α activity by repressing the production of pro-inflammatory cytokines and attenuating the transition from innate to adaptive immunity (7). Therefore, TWEAK and TNF act in a ‘Yin and Yang’ manner to modulate the transition from innate to adaptive immunity (61). Autophagy and apoptosis of macrophages play a vital role in the pathogenesis and in the host defense against M. tuberculosis (62). Herein, we show that the TWEAK-Fn14 axis-induced STIM1-ORAI1 activation, followed by increased calcium influx that activated CaMKK2 and downstream AMPK, thus promoting autophagy against early mycobacterial infection (Fig. 7). TWEAK blocker or Fn14 deficiency suppressed oxidative stress and calcium-associated autophagy, resulting in elevated mycobacterial survival. Our results also show that TWEAK-Fn14 controls mycobacterial survival by moderating oxidative stress and cytosolic Ca^2+^. We propose that the TWEAK-Fn14 axis and calcium influx could be manipulated for anti-TB therapeutic purposes. We offer a new molecular machinery to understand the association between the TWEAK-Fn14 axis, calcium influx, and mycobacterial infection.

## MATERIALS AND METHODS

### Subjects.

This prospective study was conducted at a medical center from 2019 to 2022. Active TB was proven with a positive culture or pathological findings of a tissue biopsy. A total of 20 active TB patients and 30 healthy subjects were enrolled. The Institutional Review Board of Taichung Veterans General Hospital approved this study (CE18323A), and the written consent of all participants was obtained according to the Declaration of Helsinki.

### Cells culture and mycobacteria infection.

THP-1 cells were grown in RPMI medium supplemented with 10% FBS, 1x nonessential amino acids, 100 units/mL penicillin, and 100 units/mL streptomycin, in an incubator containing 5% CO_2_ at 37°C. To induce differentiation, THP-1 cells (1.0 × 10^6^ cells) were grown in media and treated with 10 ng/mL phorbol myristate acetate (PMA; Sigma-Aldrich) overnight. The THP-1 RFP-GFP-LC3 stable cell line (Difluo hLC3) was purchased from InvivoGen and cultured on RPMI medium, according to the manufacturer’s protocol. The Mycobacterium bovis BCG (bacillus Calmette–Guérin) and M. tuberculosis H37Rv strain were cultured on Middlebrook 7H11 agar plates in an incubator containing 5% CO_2_ at 37°C. THP-1 cell-derived macrophages were infected with mycobacterial strains at an MOI of 10. At 3 h postinfection, cells were incubated with medium containing 20 mg/mL gentamicin for 1 h to kill extracellular bacteria, then washed three times with phosphate-buffered saline (PBS), and kept in the medium without antibiotics. Mycobacterial growth was determined using CFU assays. Infected cells were lysed at 72 h/indicated time postinfection. Lysates were serially diluted and plated on Middlebrook 7H11 agar plates, then incubated at 37°C to determine the CFU at 14 days. Cell viability was measured by using CellTiter-Glo Luminescent Viability assay kit (Promega) according to the manufacturer’s instructions, which determines the number of viable cells in culture by quantifying ATP.

### Transient transfection.

For Fn14 knockdown, THP-1 cell-derived macrophages were transiently transfected with 30 nM Fn14 siRNA (Dharmacon) or controls using Lipofectamine RNAiMAX transfection reagent (Thermo Fisher Scientific) according to the manufacturer’s instructions, and incubated at 37°C for 48 h. For Fn14 over-expression, THP-1 monocytes were transiently transfected with 30 ng pCMV-Fn14 or vector controls DNA using with electroporation by using Neon Transfection System Kit (Thermo Fisher Scientific) according to the manufacturer’s instructions. THP-1 monocytes of a single transfection cuvette were transferred into a single well of a 24-well plate containing 0.5 mL fresh RPMI medium. After 6 h, cells were transferred to medium containing 10 ng/mL PMA for macrophages differentiation.

### Immunoblotting.

The cells with different treatments were lysed in RIPA buffer (25 mM Tris-HCl pH 7.6, 150 mM NaCl, 1% NP-40, 1% sodium deoxycholate, and 0.1% SDS) containing a protease inhibitor cocktail (Complete, Roche). Twenty micrograms of total protein from exosome lysate were loaded and separated on a standard sodium dodecyl sulfate (SDS)-polyacrylamide gel electrophoresis (PAGE) gel and transferred to a polyvinylidene difluoride (PVDF) membrane (Millipore). The membranes were incubated with primary antibodies, followed by peroxidase-conjugated secondary antibodies. The results were detected using a charge-coupled device (CCD) camera-based imager (GE Healthcare Life Sciences) after membrane incubation with enhanced chemiluminescence (ECL) substrates (Millipore). The levels of specific protein were normalized to β-actin. ImageJ software was used for image acquisition and densitometric analysis of the immunoblots. All results were obtained in 3 independent experiments, and the data is presented as the mean ± SD. An unpaired, two-tailed Student's *t* test was performed for between-group comparisons using GraphPad Prism software version 8. All results of densitometric analysis were presented in in Supplementary File 1.

### Autophagosome maturation.

Wild type or Fn14 knockdown THP-1 cell-derived macrophages were grown on coverslips at a concentration of 1 × 10^5^ cells/well. TWEAK (100 ng/mL) was added to cells for 24 h before infection. Cells were infected with Texas Red-labeled M. bovis BCG at an MOI of 10. At 1 h postinfection, cells were incubated with medium containing 20 mg/mL gentamicin for 1 h to kill extracellular bacteria, washed three times with PBS, and incubated with LysoTracker Green (Thermo Fisher Scientific) for the final 1 h. Cells were fixed in 4% paraformaldehyde for 10 min at room temperature. Coverslips were mounted onto glass slides with mounting medium (Thermo Fisher Scientific), and images were recorded on an Olympus FV1000 laser scanning confocal microscope. Images were analyzed using FV10-ASW version 4.2 software. For quantification of the cells showing LC3-positive vesicles, approximately 50 cells were counted, and those cells with more than 20 LC3-labeled puncta were considered to have formed an autophagosome.

### Quantification of ROS production and mitochondrial membrane potential.

The levels of cytosolic/mitochondrial ROS were analyzed using the fluorescent dye dihydrorhodamine (DHR) 123 (Thermo Fisher Scientific), or MitoSOX Red (Thermo Fisher Scientific) staining and quantified by flow cytometry (FACSCanto II, BD Biosciences). Data were analyzed using the CellQuest software and expressed as the mean fluorescence intensity (MFI) of cytosolic/mitochondrial ROS.

### Quantification of intracellular calcium level.

The intracellular calcium concentration was assessed and quantified using Fluo-4 AM calcium indicator dye (Thermo Fisher Scientific) combined with flow cytometry. Briefly, naiive or Fn14 knockdown THP-1 cell-derived macrophages were incubated in HBSS-Mg^2+^ (calcium free) media and treated with anti-TWEAK monoclonal antibody for 6 h or calcium chelator BAPTA-AM (1,2-Bis(2-aminophenoxy) ethane-N, N, N’, N’-tetra acetic acid acetoxymethyl ester, 10 μM, Thermo Fisher Scientific) for 1 h. The cells were stained with Fluo-4 AM (5 μM, 1h at 37°C), then stimulated with TWEAK (100 ng/mL) or infected with Mycobacterium. The Fluo-4-AM fluorescence intensity was measured at 5 min intervals for 40 min after the activation of cells using flow cytometry.

### Flow cytometric analysis of apoptosis.

Wild type, siRNA transfection control, and Fn14 knockdown THP-1 cells were infected with M. bovis BCG at an MOI of 10 for 72 h. The apoptosis assay was measured using an Apoptosis/Necrosis assay kit (Abcam) according to the manufacturer’s protocol. ApopxinGreen indicator was used to detect the levels of membrane phosphatidylserine in apoptotic cells. Loss of plasma membrane integrity, as demonstrated by the ability of a membrane-impermeable 7-AAD to label the nucleus, represents a straightforward approach to demonstrate late-stage apoptosis and necrosis.

### Statistical analysis.

An unpaired, two-tailed Student's *t* test was used for between-group comparisons. A one-way analysis of variance (ANOVA) with the *post hoc* Bonferroni test was used for multiple comparisons. The correlation coefficient was calculated using Spearman’s correlation test. *P* values <0.05 were statistically significant and tests were performed using GraphPad Prism 8.

### Data availability.

All generated or analyzed data during this study are available within the article and Supplementary information files, or available from the authors upon request.

## References

[1] Bradfute SB, Castillo EF, Arko-Mensah J, Chauhan S, Jiang S, Mandell M, Deretic V. 2013. Autophagy as an immune effector against tuberculosis. Curr Opin Microbiol 16:355–365. doi:10.1016/j.mib.2013.05.003. 23790398 PMC3742717

[2] Ni Cheallaigh C, Keane J, Lavelle EC, Hope JC, Harris J. 2011. Autophagy in the immune response to tuberculosis: clinical perspectives. Clin Exp Immunol 164:291–300. doi:10.1111/j.1365-2249.2011.04381.x. 21438870 PMC3087924

[3] Roach DR, Bean AG, Demangel C, France MP, Briscoe H, Britton WJ. 2002. TNF regulates chemokine induction essential for cell recruitment, granuloma formation, and clearance of mycobacterial infection. J Immunol 168:4620–4627. doi:10.4049/jimmunol.168.9.4620. 11971010

[4] Moelants EA, Mortier A, Van Damme J, Proost P. 2013. Regulation of TNF-α with a focus on rheumatoid arthritis. Immunol Cell Biol 91:393–401. doi:10.1038/icb.2013.15. 23628802

[5] Winthrop KL, Baxter R, Liu L, Varley CD, Curtis JR, Baddley JW, McFarland B, Austin D, Radcliffe L, Suhler E, Choi D, Rosenbaum JT, Herrinton LJ. 2013. Mycobacterial diseases and antitumour necrosis factor therapy in USA. Ann Rheum Dis 72:37–42. doi:10.1136/annrheumdis-2011-200690. 22523429

[6] Liao TL, Lin CH, Chen YM, Chang CL, Chen HH, Chen DY. 2016. Different risk of tuberculosis and efficacy of isoniazid prophylaxis in rheumatoid arthritis patients with biologic therapy: a nationwide retrospective cohort study in Taiwan. PLoS One 11:e0153217. doi:10.1371/journal.pone.0153217. 27064275 PMC4827833

[7] Maecker H, Varfolomeev E, Kischkel F, Lawrence D, LeBlanc H, Lee W, Hurst S, Danilenko D, Li J, Filvaroff E, Yang B, Daniel D, Ashkenazi A. 2005. TWEAK attenuates the transition from innate to adaptive immunity. Cell 123:931–944. doi:10.1016/j.cell.2005.09.022. 16325585

[8] Ikner A, Ashkenazi A. 2011. TWEAK induces apoptosis through a death-signaling complex comprising receptor-interacting protein 1 (RIP1), Fas-associated death domain (FADD), and caspase-8. J Biol Chem 286:21546–21554. doi:10.1074/jbc.M110.203745. 21525013 PMC3122213

[9] Zhu C, Zhang L, Liu Z, Li C, Bai Y. 2018. TWEAK/Fn14 interaction induces proliferation and migration in human airway smooth muscle cells via activating the NF-κB pathway. J Cell Biochem 119:3528–3536. doi:10.1002/jcb.26525. 29143982

[10] Wiley SR, Cassiano L, Lofton T, Davis-Smith T, Winkles JA, Lindner V, Liu H, Daniel TO, Smith CA, Fanslow WC. 2001. A novel TNF receptor family member binds TWEAK and is implicated in angiogenesis. Immunity 15:837–846. doi:10.1016/S1074-7613(01)00232-1. 11728344

[11] Kamijo S, Nakajima A, Kamata K, Kurosawa H, Yagita H, Okumura K. 2008. Involvement of TWEAK/Fn14 interaction in the synovial inflammation of RA. Rheumatology (Oxford) 47:442–450. doi:10.1093/rheumatology/ken006. 18310134

[12] Chen DY, Chen YM, Lin CF, Lo CM, Liu HJ, Liao TL. 2020. MicroRNA-889 Inhibits autophagy to maintain mycobacterial survival in patients with latent tuberculosis infection by targeting TWEAK. mBio 11:e03045-19. doi:10.1128/mBio.03045-19. 31992621 PMC6989109

[13] van Kuijk AW, Wijbrandts CA, Vinkenoog M, Zheng TS, Reedquist KA, Tak PP. 2010. TWEAK and its receptor Fn14 in the synovium of patients with rheumatoid arthritis compared to psoriatic arthritis and its response to tumour necrosis factor blockade. Ann Rheum Dis 69:301–304. doi:10.1136/ard.2008.090548. 19147618 PMC2789939

[14] Michaelson JS, Wisniacki N, Burkly LC, Putterman C. 2012. Role of TWEAK in lupus nephritis: a bench-to-bedside review. J Autoimmun 39:130–142. doi:10.1016/j.jaut.2012.05.003. 22727560 PMC3428508

[15] Winkles JA. 2008. The TWEAK–Fn14 cytokine–receptor axis: discovery, biology and therapeutic targeting. Nat Rev Drug Discov 7:411–425. doi:10.1038/nrd2488. 18404150 PMC3018765

[16] Filomeni G, De Zio D, Cecconi F. 2015. Oxidative stress and autophagy: the clash between damage and metabolic needs. Cell Death Differ 22:377–388. doi:10.1038/cdd.2014.150. 25257172 PMC4326572

[17] Madrigal-Matute J, Fernandez-Laso V, Sastre C, Llamas-Granda P, Egido J, Martin-Ventura JL, Zalba G, Blanco-Colio LM. 2015. TWEAK/Fn14 interaction promotes oxidative stress through NADPH oxidase activation in macrophages. Cardiovasc Res 108:139–147. doi:10.1093/cvr/cvv204. 26224570

[18] Liu H, Peng H, Xiang H, Guo L, Chen R, Zhao S, Chen W, Chen P, Lu H, Chen S. 2018. TWEAK/Fn14 promotes oxidative stress through AMPK/PGC1alpha/MnSOD signaling pathway in endothelial cells. Mol Med Rep 17:1998–2004. 29257217 10.3892/mmr.2017.8090

[19] Chinopoulos C, Adam-Vizi V. 2006. Calcium, mitochondria and oxidative stress in neuronal pathology. Novel aspects of an enduring theme. FEBS J 273:433–450. doi:10.1111/j.1742-4658.2005.05103.x. 16420469

[20] Hurley RL, Anderson KA, Franzone JM, Kemp BE, Means AR, Witters LA. 2005. The Ca^2+^/calmodulin-dependent protein kinase kinases are AMP-activated protein kinase kinases. J Biol Chem 280:29060–29066. doi:10.1074/jbc.M503824200. 15980064

[21] Lee HJ, Ko HJ, Song DK, Jung YJ. 2018. Lysophosphatidylcholine promotes phagosome maturation and regulates inflammatory mediator production through the protein kinase A–phosphatidylinositol 3 kinase–p38 mitogen-activated protein kinase signaling pathway during *Mycobacterium tuberculosis* infection in mouse macrophages. Front Immunol 9:920. doi:10.3389/fimmu.2018.00920. 29755479 PMC5934435

[22] Croft M, Siegel RM. 2017. Beyond TNF: TNF superfamily cytokines as targets for the treatment of rheumatic diseases. Nat Rev Rheumatol 13:217–233. doi:10.1038/nrrheum.2017.22. 28275260 PMC5486401

[23] Harris J, Hope JC, Keane J. 2008. Tumor necrosis factor blockers influence macrophage responses to *Mycobacterium tuberculosis*. J Infect Dis 198:1842–1850. doi:10.1086/593174. 18954258

[24] Maiuri MC, Zalckvar E, Kimchi A, Kroemer G. 2007. Self-eating and self-killing: crosstalk between autophagy and apoptosis. Nat Rev Mol Cell Biol 8:741–752. doi:10.1038/nrm2239. 17717517

[25] Riendeau CJ, Kornfeld H. 2003. THP-1 cell apoptosis in response to Mycobacterial infection. Infect Immun 71:254–259. doi:10.1128/IAI.71.1.254-259.2003. 12496173 PMC143334

[26] Nakayama M, Ishidoh K, Kayagaki N, Kojima Y, Yamaguchi N, Nakano H, Kominami E, Okumura K, Yagita H. 2002. Multiple pathways of TWEAK-induced cell death. J Immunol 168:734–743. doi:10.4049/jimmunol.168.2.734. 11777967

[27] Gottlieb E, Armour SM, Harris MH, Thompson CB. 2003. Mitochondrial membrane potential regulates matrix configuration and cytochrome c release during apoptosis. Cell Death Differ 10:709–717. doi:10.1038/sj.cdd.4401231. 12761579

[28] Duchen MR. 2000. Mitochondria and calcium: from cell signalling to cell death. J Physiol 529 Pt 1:57–68. doi:10.1111/j.1469-7793.2000.00057.x. 11080251 PMC2270168

[29] Romagnoli A, Etna MP, Giacomini E, Pardini M, Remoli ME, Corazzari M, Falasca L, Goletti D, Gafa V, Simeone R, Delogu G, Piacentini M, Brosch R, Fimia GM, Coccia EM. 2012. ESX-1 dependent impairment of autophagic flux by *Mycobacterium tuberculosis* in human dendritic cells. Autophagy 8:1357–1370. doi:10.4161/auto.20881. 22885411 PMC3442882

[30] Brosch R, Gordon SV, Garnier T, Eiglmeier K, Frigui W, Valenti P, Dos Santos S, Duthoy S, Lacroix C, Garcia-Pelayo C, Inwald JK, Golby P, Garcia JN, Hewinson RG, Behr MA, Quail MA, Churcher C, Barrell BG, Parkhill J, Cole ST. 2007. Genome plasticity of BCG and impact on vaccine efficacy. Proc Natl Acad Sci USA 104:5596–5601. doi:10.1073/pnas.0700869104. 17372194 PMC1838518

[31] Høyer-Hansen M, Bastholm L, Szyniarowski P, Campanella M, Szabadkai G, Farkas T, Bianchi K, Fehrenbacher N, Elling F, Rizzuto R, Mathiasen IS, Jäättelä M. 2007. Control of macroautophagy by calcium, calmodulin-dependent kinase kinase-beta, and Bcl-2. Mol Cell 25:193–205. doi:10.1016/j.molcel.2006.12.009. 17244528

[32] Smaili SS, Pereira GJS, Costa MM, Rocha KK, Rodrigues L, do Carmo LG, Hirata H, Hsu Y-T. 2013. The role of calcium stores in apoptosis and autophagy. Curr Mol Med 13:252–265. doi:10.2174/156652413804810772. 23228221

[33] Yang J, Yu J, Li D, Yu S, Ke J, Wang L, Wang Y, Qiu Y, Gao X, Zhang J, Huang L. 2017. Store-operated calcium entry-activated autophagy protects EPC proliferation via the CAMKK2-MTOR pathway in ox-LDL exposure. Autophagy 13:82–98. doi:10.1080/15548627.2016.1245261. 27791458 PMC5240837

[34] Francis RJ, Butler RE, Stewart GR. 2014. *Mycobacterium tuberculosis* ESAT-6 is a leukocidin causing Ca^2+^ influx, necrosis and neutrophil extracellular trap formation. Cell Death Dis 5:e1474. doi:10.1038/cddis.2014.394. 25321481 PMC4237235

[35] Jayachandran R, Sundaramurthy V, Combaluzier B, Mueller P, Korf H, Huygen K, Miyazaki T, Albrecht I, Massner J, Pieters J. 2007. Survival of mycobacteria in macrophages is mediated by coronin 1-dependent activation of calcineurin. Cell 130:37–50. doi:10.1016/j.cell.2007.04.043. 17632055

[36] Malik ZA, Denning GM, Kusner DJ. 2000. Inhibition of Ca^2+^ signaling by *Mycobacterium tuberculosis* is associated with reduced phagosome–lysosome fusion and increased survival within human macrophages. J Exp Med 191:287–302. doi:10.1084/jem.191.2.287. 10637273 PMC2195750

[37] Liu F, Chen J, Wang P, Li H, Zhou Y, Liu H, Liu Z, Zheng R, Wang L, Yang H, Cui Z, Wang F, Huang X, Wang J, Sha W, Xiao H, Ge B. 2018. MicroRNA-27a controls the intracellular survival of *Mycobacterium tuberculosis* by regulating calcium-associated autophagy. Nat Commun 9:4295. doi:10.1038/s41467-018-06836-4. 30327467 PMC6191460

[38] Chandra P, Grigsby SJ, Philips JA. 2022. Immune evasion and provocation by *Mycobacterium tuberculosis*. Nat Rev Microbiol 1–17. doi:10.1038/s41579-022-00763-4. PMC9310001 35879556

[39] Li H, Zhou X, Huang Y, Liao B, Cheng L, Ren B. 2020. Reactive oxygen species in pathogen clearance: the killing mechanisms, the adaption response, and the side effects. Front Microbiol 11:622534. 33613470 10.3389/fmicb.2020.622534 PMC7889972

[40] Martinez J, Malireddi RKS, Lu Q, Cunha LD, Pelletier S, Gingras S, Orchard R, Guan J-L, Tan H, Peng J, Kanneganti T-D, Virgin HW, Green DR. 2015. Molecular characterization of LC3-associated phagocytosis reveals distinct roles for Rubicon, NOX2 and autophagy proteins. Nat Cell Biol 17:893–906. doi:10.1038/ncb3192. 26098576 PMC4612372

[41] Samidurai M, Tarale P, Janarthanam C, Estrada CG, Gordon R, Zenitsky G, Jin H, Anantharam V, Kanthasamy AG, Kanthasamy A. 2020. Tumor necrosis factor-like weak inducer of apoptosis (TWEAK) enhances activation of STAT3/NLRC4 inflammasome signaling axis through PKCδ in astrocytes: implications for Parkinson's Disease Cells 9:1831. doi:10.3390/cells9081831. 32759670 PMC7464730

[42] Roca FJ, Ramakrishnan L. 2013. TNF dually mediates resistance and susceptibility to mycobacteria via mitochondrial reactive oxygen species. Cell 153:521–534. doi:10.1016/j.cell.2013.03.022. 23582643 PMC3790588

[43] Chaudhari N, Talwar P, Parimisetty A, Lefebvre d’Hellencourt C, Ravanan P. 2014. A molecular web: endoplasmic reticulum stress, inflammation, and oxidative stress. Front Cell Neurosci 8:213. doi:10.3389/fncel.2014.00213. 25120434 PMC4114208

[44] Peng TI, Jou MJ. 2010. Oxidative stress caused by mitochondrial calcium overload. Ann N Y Acad Sci 1201:183–188. doi:10.1111/j.1749-6632.2010.05634.x. 20649555

[45] Hempel N, Trebak M. 2017. Crosstalk between calcium and reactive oxygen species signaling in cancer. Cell Calcium 63:70–96. doi:10.1016/j.ceca.2017.01.007. 28143649 PMC5466514

[46] Prakriya M, Lewis RS. 2015. Store-operated calcium channels. Physiol Rev 95:1383–1436. doi:10.1152/physrev.00020.2014. 26400989 PMC4600950

[47] Bhardwaj R, Hediger MA, Demaurex N. 2016. Redox modulation of STIM-ORAI signaling. Cell Calcium 60:142–152. doi:10.1016/j.ceca.2016.03.006. 27041216

[48] Kahlfuss S, Kaufmann U, Concepcion AR, Noyer L, Raphael D, Vaeth M, Yang J, Pancholi P, Maus M, Muller J, Kozhaya L, Khodadadi-Jamayran A, Sun Z, Shaw P, Unutmaz D, Stathopulos PB, Feist C, Cameron SB, Turvey SE, Feske S. 2020. STIM1-mediated calcium influx controls antifungal immunity and the metabolic function of non-pathogenic Th17 cells. EMBO Mol Med 12:e11592. doi:10.15252/emmm.201911592. 32609955 PMC7411566

[49] Liu C-C, Miao Y, Chen R-L, Zhang Y-Q, Wu H, Yang S-M, Shang L-Q. 2021. STIM1 mediates IAV-induced inflammation of lung epithelial cells by regulating NLRP3 and inflammasome activation via targeting miR-223. Life Sci 266:118845. doi:10.1016/j.lfs.2020.118845. 33278394

[50] Desvignes L, Weidinger C, Shaw P, Vaeth M, Ribierre T, Liu M, Fergus T, Kozhaya L, McVoy L, Unutmaz D, Ernst JD, Feske S. 2015. STIM1 controls T cell–mediated immune regulation and inflammation in chronic infection. J Clin Invest 125:2347–2362. doi:10.1172/JCI80273. 25938788 PMC4518689

[51] Hawkins BJ, Irrinki KM, Mallilankaraman K, Lien Y-C, Wang Y, Bhanumathy CD, Subbiah R, Ritchie MF, Soboloff J, Baba Y, Kurosaki T, Joseph SK, Gill DL, Madesh M. 2010. S-glutathionylation activates STIM1 and alters mitochondrial homeostasis. J Cell Biol 190:391–405. doi:10.1083/jcb.201004152. 20679432 PMC2922639

[52] Gurunathan S, Winkles JA, Ghosh S, Hayden MS. 2014. Regulation of fibroblast growth factor-inducible 14 (Fn14) expression levels via ligand-independent lysosomal degradation. J Biol Chem 289:12976–12988. doi:10.1074/jbc.M114.563478. 24652288 PMC4036313

[53] Blanco-Colio L. 2014. TWEAK/Fn14 Axis: A Promising Target for the Treatment of Cardiovascular Diseases. Front Immunol 5:3. doi:10.3389/fimmu.2014.00003. 24478772 PMC3895871

[54] Zhao Z, Burkly LC, Campbell S, Schwartz N, Molano A, Choudhury A, Eisenberg RA, Michaelson JS, Putterman C. 2007. TWEAK/Fn14 interactions are instrumental in the pathogenesis of nephritis in the chronic graft-versus-host model of systemic lupus erythematosus. J Immunol 179:7949–7958. doi:10.4049/jimmunol.179.11.7949. 18025243

[55] Cheng E, Armstrong C, Galisteo R, Winkles J. 2013. TWEAK/Fn14 axis-targeted therapeutics: moving basic science discoveries to the clinic Front Immunol 4:473. doi:10.3389/fimmu.2013.00473. 24391646 PMC3870272

[56] Butler RE, Brodin P, Jang J, Jang M-S, Robertson BD, Gicquel B, Stewart GR. 2012. The balance of apoptotic and necrotic cell death in *Mycobacterium tuberculosis* infected macrophages is not dependent on bacterial virulence. PLoS One 7:e47573. doi:10.1371/journal.pone.0047573. 23118880 PMC3484146

[57] Roca FJ, Whitworth LJ, Redmond S, Jones AA, Ramakrishnan L. 2019. TNF induces pathogenic programmed macrophage necrosis in tuberculosis through a mitochondrial-lysosomal-endoplasmic reticulum circuit. Cell 178:1344–1361. doi:10.1016/j.cell.2019.08.004. 31474371 PMC6736209

[58] Martin-Sanchez D, Fontecha-Barriuso M, Carrasco S, Sanchez-Niño MD, Mässenhausen Av, Linkermann A, Cannata-Ortiz P, Ruiz-Ortega M, Egido J, Ortiz A, Sanz AB. 2018. TWEAK and RIPK1 mediate a second wave of cell death during AKI. Proc Natl Acad Sci USA 115:4182–4187. doi:10.1073/pnas.1716578115. 29588419 PMC5910825

[59] O'Sullivan MP, O'Leary S, Kelly DM, Keane J. 2007. A caspase-independent pathway mediates macrophage cell death in response to *Mycobacterium tuberculosis* infection. Infect Immun 75:1984–1993. doi:10.1128/IAI.01107-06. 17283090 PMC1865710

[60] Polek TC, Talpaz M, Darnay BG, Spivak-Kroizman T. 2003. TWEAK mediates signal transduction and differentiation of RAW264.7 cells in the absence of Fn14/TweakR: EVIDENCE FOR A SECOND TWEAK RECEPTOR*. J Biol Chem 278:32317–32323. doi:10.1074/jbc.M302518200. 12794080

[61] Bell E. 2006. TWEAK and TNF: yin and yang in innate immunity. Nat Rev Immunol 6:91–91. doi:10.1038/nri1790.

[62] Lam A, Prabhu R, Gross CM, Riesenberg LA, Singh V, Aggarwal S. 2017. Role of apoptosis and autophagy in tuberculosis. Am J Physiol Lung Cell Mol Physiol 313:L218–L229. doi:10.1152/ajplung.00162.2017. 28495854 PMC5582934

