# Supplementary material for: TWEAK-Fn14 Axis Induces Calcium-Associated Autophagy and Cell Death To Control Mycobacterial Survival in Macrophages
Source: Microbiol Spectr. 2022 Nov 2;10(6):e03172-22. doi: 10.1128/spectrum.03172-22 (PMC9769850; doi:10.1128/spectrum.03172-22)
Supplement: Supplemental file 1 — Supplemental material. Download spectrum.03172-22-s0001.pdf, PDF file, 1.7 MB [file spectrum.03172-22-s0001.pdf]

Supplementary information

TWEAK-Fn14 axis induces calcium-associated autophagy and cell death to control mycobacterial survival in macrophages

Yi-Ming Chen[1-4], Po-Yu Liu[2,3,5], Kuo-Tung Tang[4], Hung-Jen Liu[2,3,6,7], Tsai-Ling Liao[1-3*]

[1]Department of Medical Research, Taichung Veterans General Hospital, Taichung, Taiwan
[2]Rong Hsing Research Center for Translational Medicine, National Chung Hsing University, Taichung 402, Taiwan
[3]Ph.D. Program in Translational Medicine, National Chung Hsing University, Taichung 402, Taiwan
[4]Division of Allergy, Immunology and Rheumatology, Department of Internal Medicine, Taichung Veterans General Hospital, Taichung, Taiwan
[5]Division of Infection, Department of Internal Medicine, Taichung Veterans General Hospital, Taichung, Taiwan
[6]Institute of Molecular Biology, National Chung Hsing University, Taichung 402, Taiwan
[7]The iEGG and Animal Biotechnology Center, National Chung Hsing University, Taichung 402, Taiwan

Corresponding author and address reprint requests:

Dr. Tsai-Ling Liao, Department of Medical Research, Taichung Veterans General Hospital, Taichung, Taiwan

Address: No.1650, Sec.4, Taiwan Boulevard, Xitun Dist., Taichung City 40705, Taiwan
Tel.: 886-4-23592525, extension 4020; Fax: 886-4-23592705
Email: tlliao@vghtc.gov.tw

Supplementary Materials

Reagent or Resource	Source	Identifier (Cat No.)
Antibodies		
Mouse anti-β-actin antibodies	Santa Cruz	sc-47778
Mouse anti-IP3RII antibody	Santa Cruz	sc-398434
Mouse anti-CHOP antibody	Cell Signaling Technology	#2895
Mouse anti-ORAI1 antibody	Santa Cruz	sc-377281
Mouse anti-STIM1 antibody	Santa Cruz	sc-166840
Mouse anti-TRPM7 antibody	Santa Cruz	sc-271099
Rabbit anti-AMPK antibody	Cell Signaling Technology	#5832
Rabbit anti-pAMPK(T172) antibody	Cell Signaling Technology	#2535
Rabbit anti-Atg5 antibody	Cell Signaling Technology	#12994
Rabbit anti-Beclin-1 antibody	Cell Signaling Technology	#3738
Rabbit anti-BiP antibody	Cell Signaling Technology	#3177
Rabbit anti-cleaved caspase-3 antibody	Cell Signaling Technology	#9661
Rabbit anti-cleaved caspase-8 antibody	Cell Signaling Technology	#9496
Rabbit anti-Fn14 antibody	Cell Signaling Technology	#4403
Rabbit anti-LC3B antibody	Cell Signaling Technology	#2775
Rabbit anti-pCaMKK2 (S511) antibody	Cell Signaling Technology	#12818
Rabbit anti-pMLKL (S358) antibody	Cell Signaling Technology	#91689
Rabbit anti-pRIPK1 (S166) antibody	Cell Signaling Technology	#65746
Rabbit anti-pRIPK3 (S227) antibody	Cell Signaling Technology	#13526
Rabbit anti-pULK1(S555)	Cell Signaling	#5869

antibody	Technology	
Rabbit anti-ULK1 antibody	Cell Signaling Technology	#6439
Rabbit anti-p62 antibody	Cell Signaling Technology	#8025
Rabbit anti-TWEAK antibody	Abcam	ab37170
Alexa Fluor 488 Goat anti-Rabbit IgG	Thermo Fisher Scientific	A11008
Anti-mouse IgG, HRP-linked antibody	Cell Signaling Technology	#7076
Anti-rabbit IgG, HRP-linked antibody	Cell Signaling Technology	#7074
Chemicals, Peptides, and Recombinant Proteins		
3-methyladenine (3-MA)	Sigma-Aldrich	M9281
A23187 calcium ionophore	Sigma-Aldrich	C7522
BAPTA-AM	Sigma-Aldrich	A1076
bepridil hydrochloride	Sigma-Aldrich	B5016
BTP2	Sigma-Aldrich	Y4895
dihydrorhodamine 123	Thermo Fisher Scientific	D23806
dimethyl sulfoxide	Sigma-Aldrich	D8418
diphenyleneiodonium	Sigma-Aldrich	D2926
LysoTracker Green	Thermo Fisher Scientific	L7526
Fluo 4-AM	Thermo Fisher Scientific	F14217
JC-1	Abcam	ab113850
Mito-ID	Enzo Life Sciences	ENZ-51018
MitoSOX Red	Thermo Fisher Scientific	M36008
MitoTEMPO	Sigma-Aldrich	SML0737
phorbol myristate acetate	Sigma-Aldrich	P1585
Recombinant human TWEAK	PeproTech	310-06
Texas Red-X	Thermo Fisher Scientific	T20175
Trizol reagent	Thermo Fisher	15596018

	Scientific	
Z-VAD-FMK	Sigma-Aldrich	V116
Cell lines		
THP-1 Difluo^{TM} hLC3 cells	InvivoGen	THPDF-HLC3
Others		
Lipofectamine RNAiMAX Transfection Reagent	Thermo Fisher Scientific	13778150
Fn14 TaqMan Gene Expression Assays	Thermo Fisher Scientific	Hs00171993_m1
Human TWEAK Instant ELISA kit	Thermo Fisher Scientific	BMS2006INST
On TARGETplus SMARTpool siFn14	Dharmacon	L-010661-00-0005
Apoptosis/ Necrosis Assay kit	Abcam	ab176749
CellTiter-Glo Luminescent Cell Viability Assay	Promega	G7570

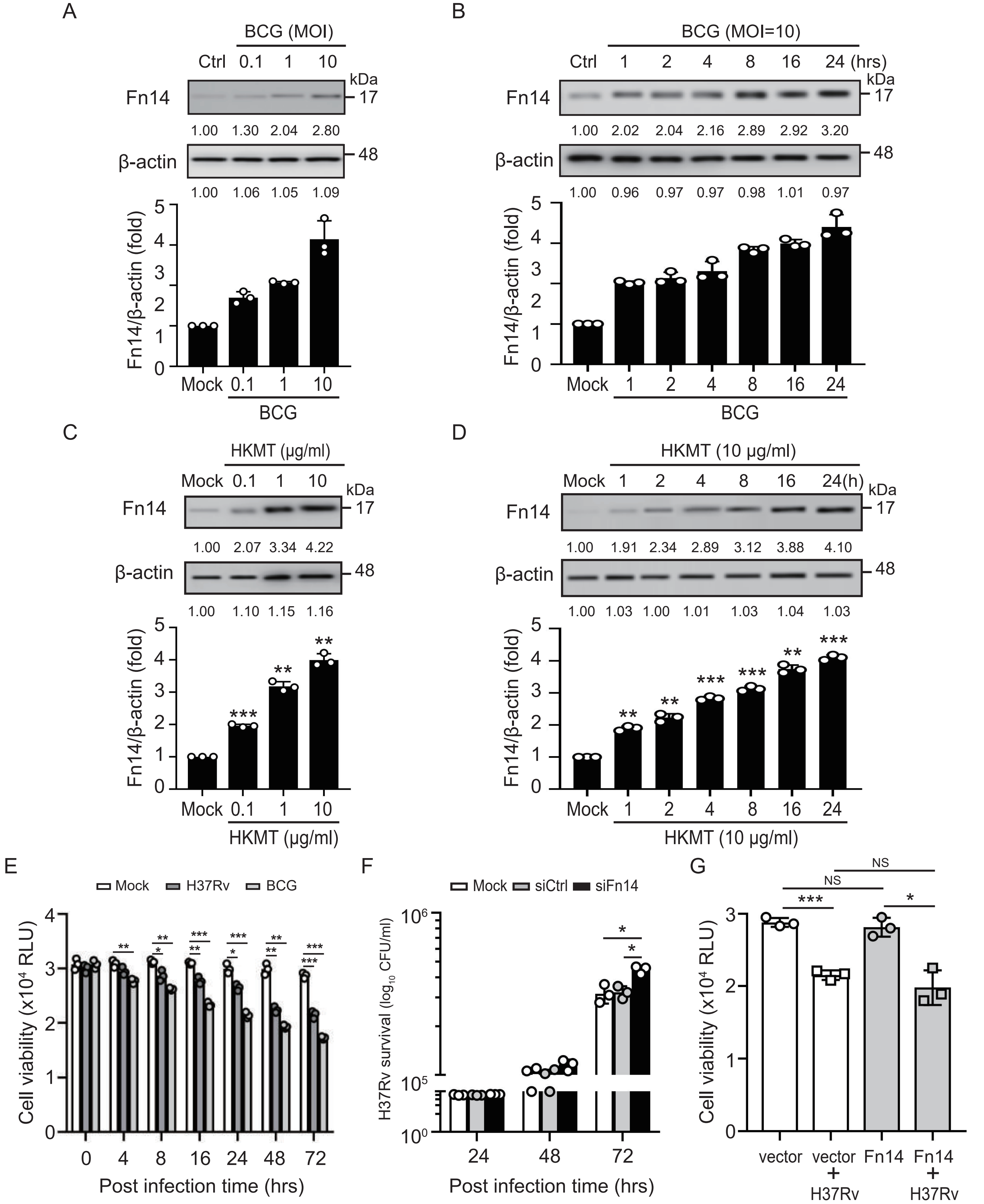

Supplementary Figure S1 (A to D) Fn14 expression is increased after (A,B) BCG infection or (C,D) heat-killed *M. tuberculosis* (HKMT) treatment in a dose- and time- dependent manner. (E) The cell viability of THP-1 cell-derived macrophages with H37Rv or BCG infection at a MOI of 10. (F) The time kinetic of H37Rv proliferation in Fn14 knockdown cells. (G)The cell viability of vector-transfected or Fn14 over-expressing cells with or without H37Rv infection. Immunoblots were quantitated by densitometric analysis using ImageJ software and normalized to β-actin. Numbers below each lane are relative fold of the control level of a specific protein in mock-treated cells. All experiments were performed in triplicate and data are presented as the mean±SD. * *P*<0.05, ** *P*<0.01, *** *P*<0.005. NS, no significant.

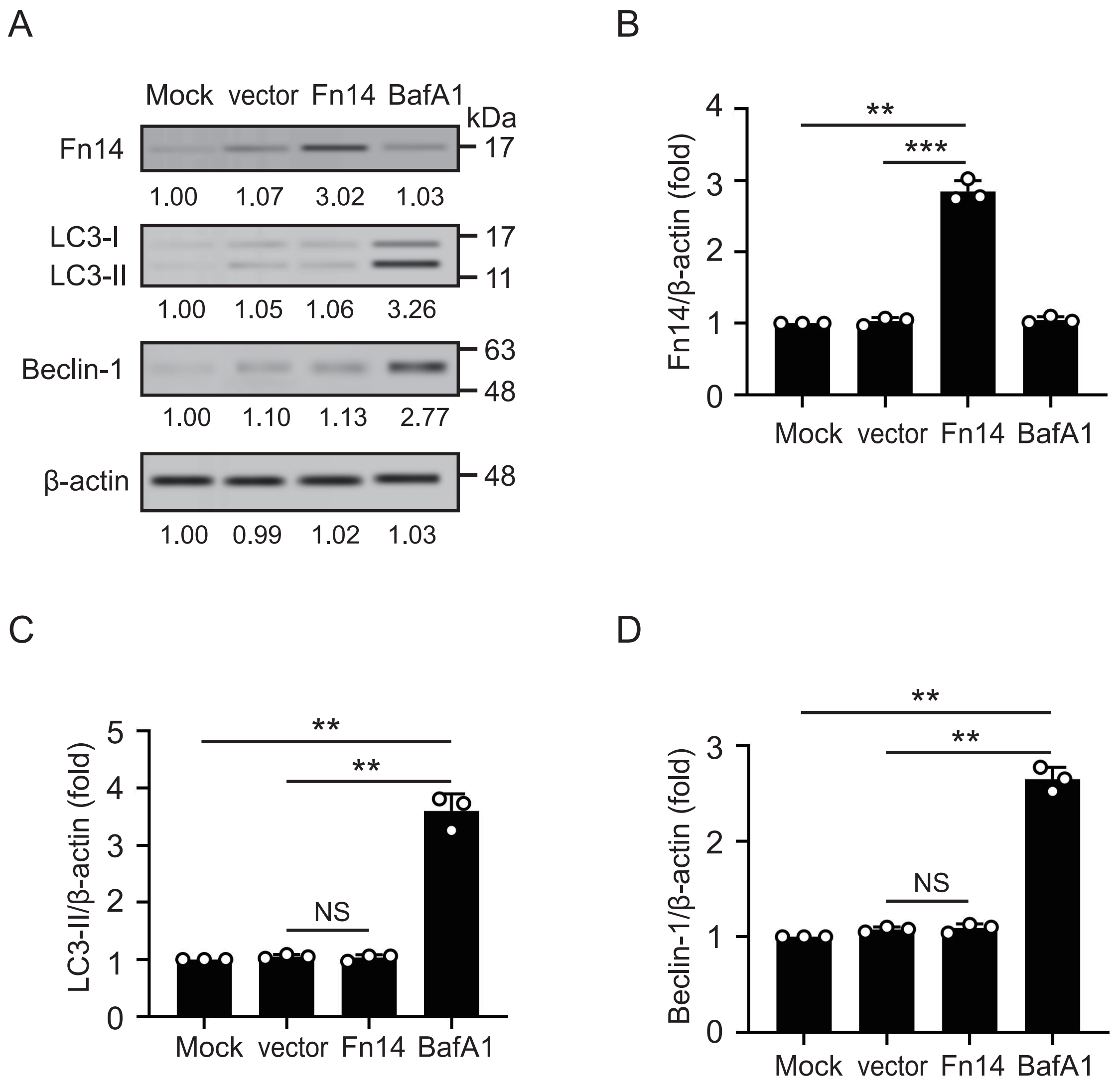

Supplementary Figure S2 THP-1 cell-derived macrophages were transfected with pCMV-Fn14 to overexpress Fn14. After 24 h, (A) the levels of Fn14, LC3, Beclin-1, and β-actin were detected by immunoblotting. The bafilomycin A1 (BafA1) treatment was used as positive control. Immunoblots were quantitated by densitometric analysis using ImageJ software and normalized to β-actin. Numbers below each lane are relative fold of the control level of a specific protein in mock-treated cells. The relative levels of (B) Fn14, (C) LC3-II, and (D) Beclin-1 were calculated, respectively. All experiments were performed in triplicate and data are presented as the mean±SD. ** P<0.01, *** P<0.005. NS, no significant.

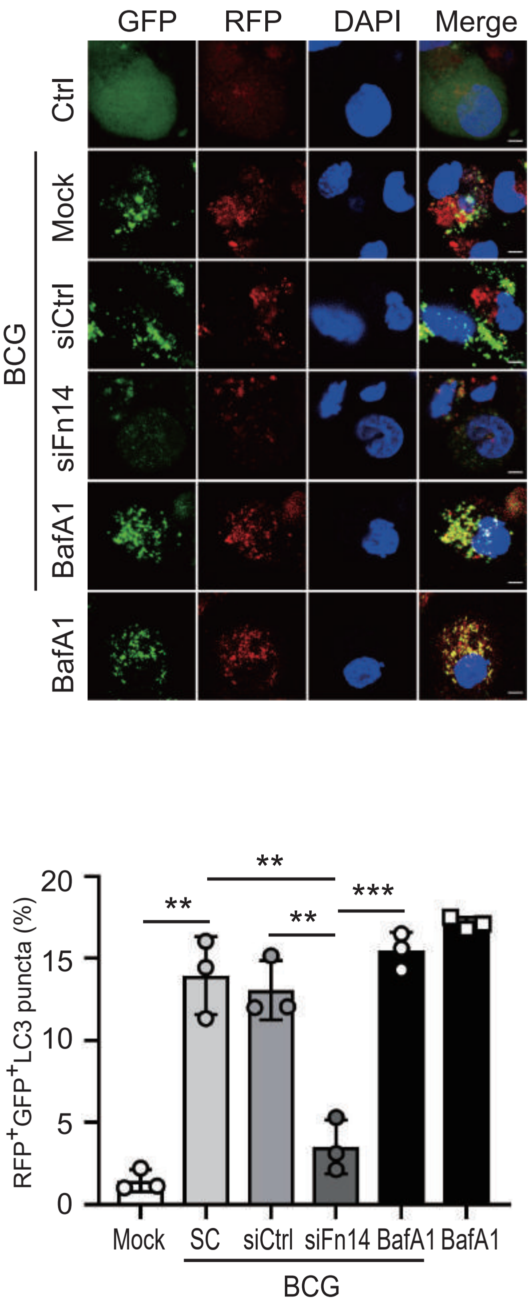

Supplementary Figure S3 THP-1 cells stably expressing RFP-GFP-LC3 fusion protein were transfected with Fn14 siRNA to knockdown Fn14. After 24 h, cells were infected with *M. bovis* BCG at a MOI of 10 for 24 h. The RFP-GFP-LC3 puncta were detected by confocal microscopy (upper panel) and quantified (lower panel). RPMI medium were used as solvent control (SC). Bafilomycin A1 (BafA1, 100nM) treatment were used as positive control. All experiments were performed in triplicate and data are presented as the mean±SD.***P*<0.01, ****P*<0.005.

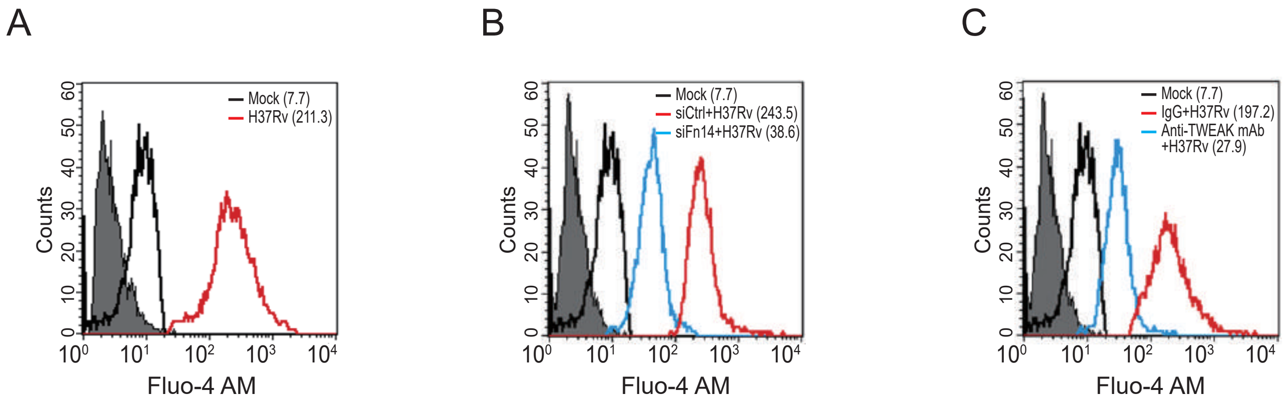

Supplementary Figure S4 THP-1 cell-derived macrophages were transfected with siFn14 or treated with anti-TWEAK monoclonal antibody for 6h. The cells were stained with Flou-4 AM (5 μM, 1h at 37°C), then infected with *M. tuberculosis* H37Rv at MOI of 1 (20 min). The calcium mobilization in Fluo-4 AM loading cells was detected and quantified using flow cytometry. (A) H37Rv infection induced increased levels of cytosolic calcium in THP-1-derived macrophages. This effect was diminished when (B) intracellular Fn14 was knocked down or (C) in the presence of TWEAK blocker.

Supplementary File 1. Densitometric analysis of immunoblot results presented in this study. Immunoblots were quantitated by densitometric analysis using ImageJ software and normalized to β-actin. Numbers below each lane are relative fold of the control level of a specific protein in mock-treated cells. All results were obtained in three independent experiments, and the data is presented as the mean±SD. *$P<0.05$, ** $P<0.01$, *** $P<0.005$.

Fig. 2A

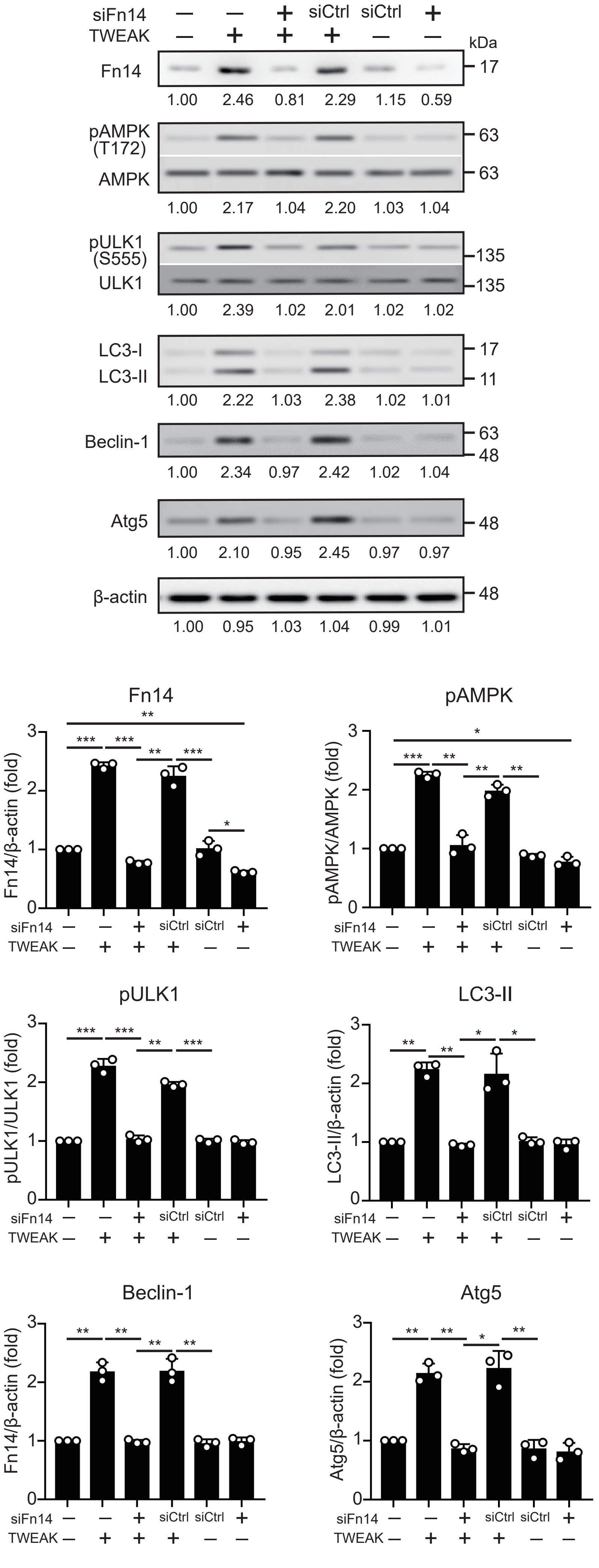

siFn14
TWEAK
siCtrl siCtrl
kDa
Fn14
17
1.00 2.46 0.81 2.29 1.15 0.59
pAMPK (T172)
AMPK
63
63
1.00 2.17 1.04 2.20 1.03 1.04
pULK1 (S555)
ULK1
135
135
1.00 2.39 1.02 2.01 1.02 1.02
LC3-I
LC3-II
17
11
1.00 2.22 1.03 2.38 1.02 1.01
Beclin-1
63
48
1.00 2.34 0.97 2.42 1.02 1.04
Atg5
48
1.00 2.10 0.95 2.45 0.97 0.97
β-actin
48
1.00 0.95 1.03 1.04 0.99 1.01
Fn14
Fn14/β-actin (fold)
pAMPK
pAMPK/AMPK (fold)
pULK1
pULK1/ULK1 (fold)
LC3-II
LC3-II/β-actin (fold)
Beclin-1
Fn14/β-actin (fold)
Atg5
Atg5/β-actin (fold)

Fig. 2F

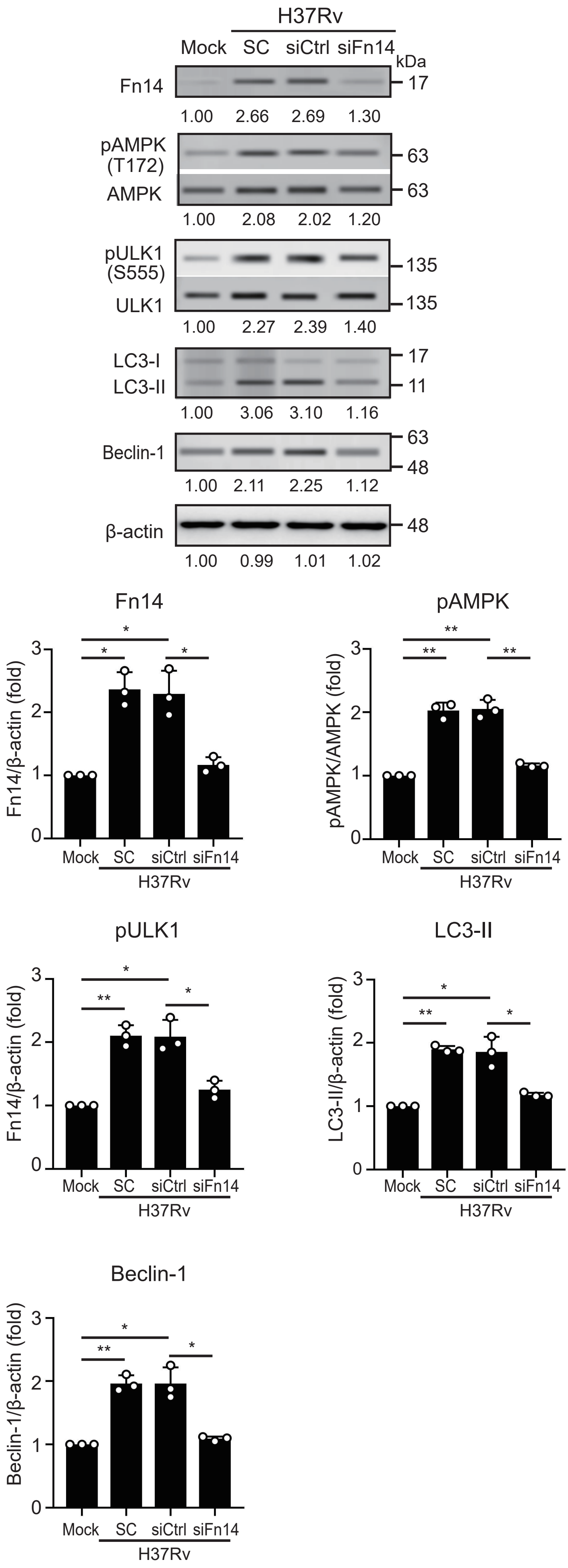
H37Rv
Mock SC siCtrl siFn14
kDa
Fn14
17
1.00 2.66 2.69 1.30
pAMPK (T172)
63
AMPK
63
1.00 2.08 2.02 1.20
pULK1 (S555)
135
ULK1
135
1.00 2.27 2.39 1.40
LC3-I
17
LC3-II
11
1.00 3.06 3.10 1.16
Beclin-1
63
48
1.00 2.11 2.25 1.12
β-actin
48
1.00 0.99 1.01 1.02
Fn14
Fn14/β-actin (fold)
pAMPK
pAMPK/AMPK (fold)
pULK1
Fn14/β-actin (fold)
LC3-II
LC3-II/β-actin (fold)
Beclin-1
Beclin-1/β-actin (fold)
Mock SC siCtrl siFn14
H37Rv

Fig. 3C

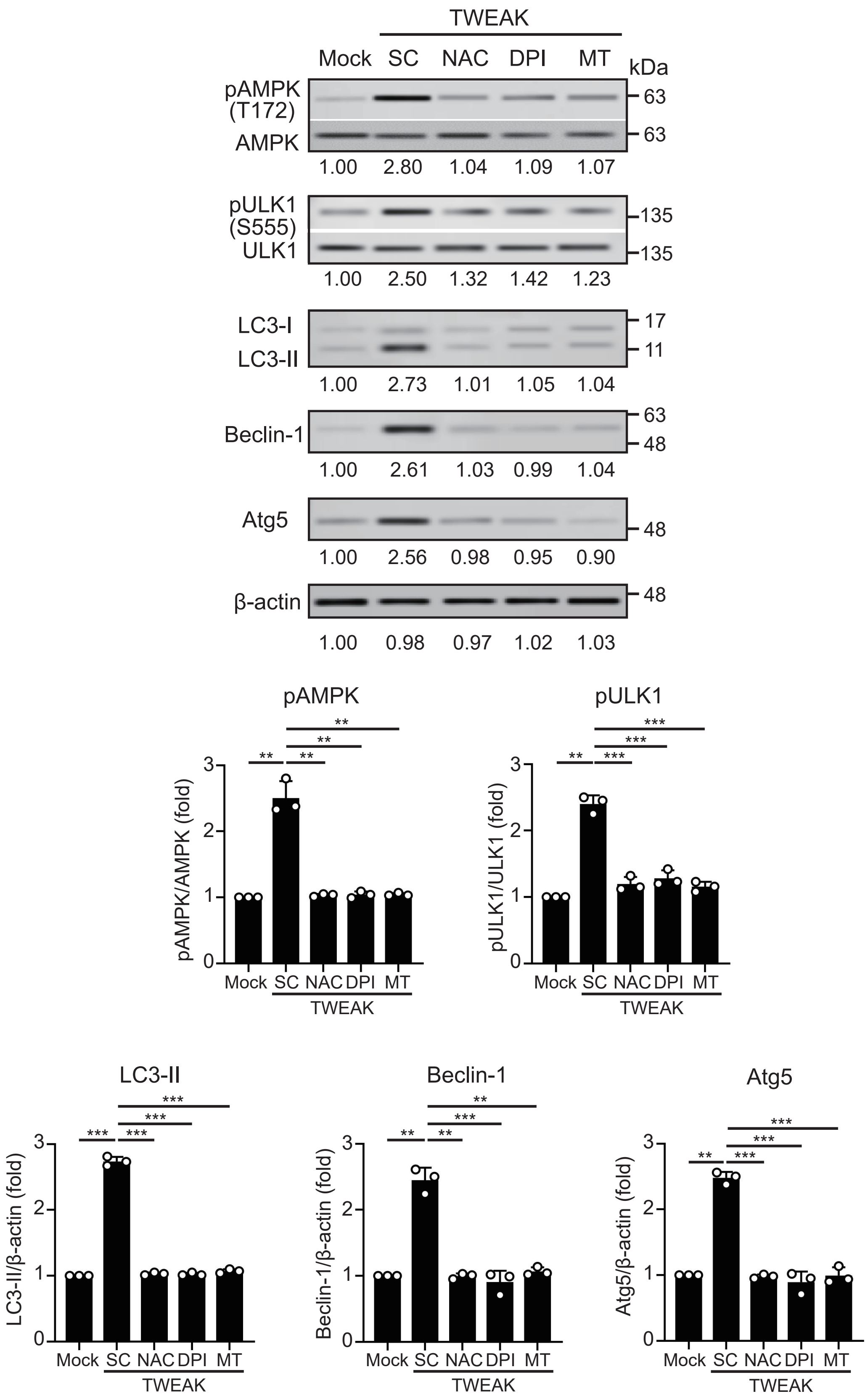

TWEAK
Mock SC NAC DPI MT
kDa
pAMPK (T172)
AMPK
63
63
1.00 2.80 1.04 1.09 1.07
pULK1 (S555)
ULK1
135
135
1.00 2.50 1.32 1.42 1.23
LC3-I
LC3-II
17
11
1.00 2.73 1.01 1.05 1.04
Beclin-1
63
48
1.00 2.61 1.03 0.99 1.04
Atg5
48
1.00 2.56 0.98 0.95 0.90
β-actin
48
1.00 0.98 0.97 1.02 1.03
pAMPK
pAMPK/AMPK (fold)
pULK1
pULK1/ULK1 (fold)
LC3-II
LC3-II/β-actin (fold)
Beclin-1
Beclin-1/β-actin (fold)
Atg5
Atg5/β-actin (fold)
Mock SC NAC DPI MT
TWEAK

Fig. 4A

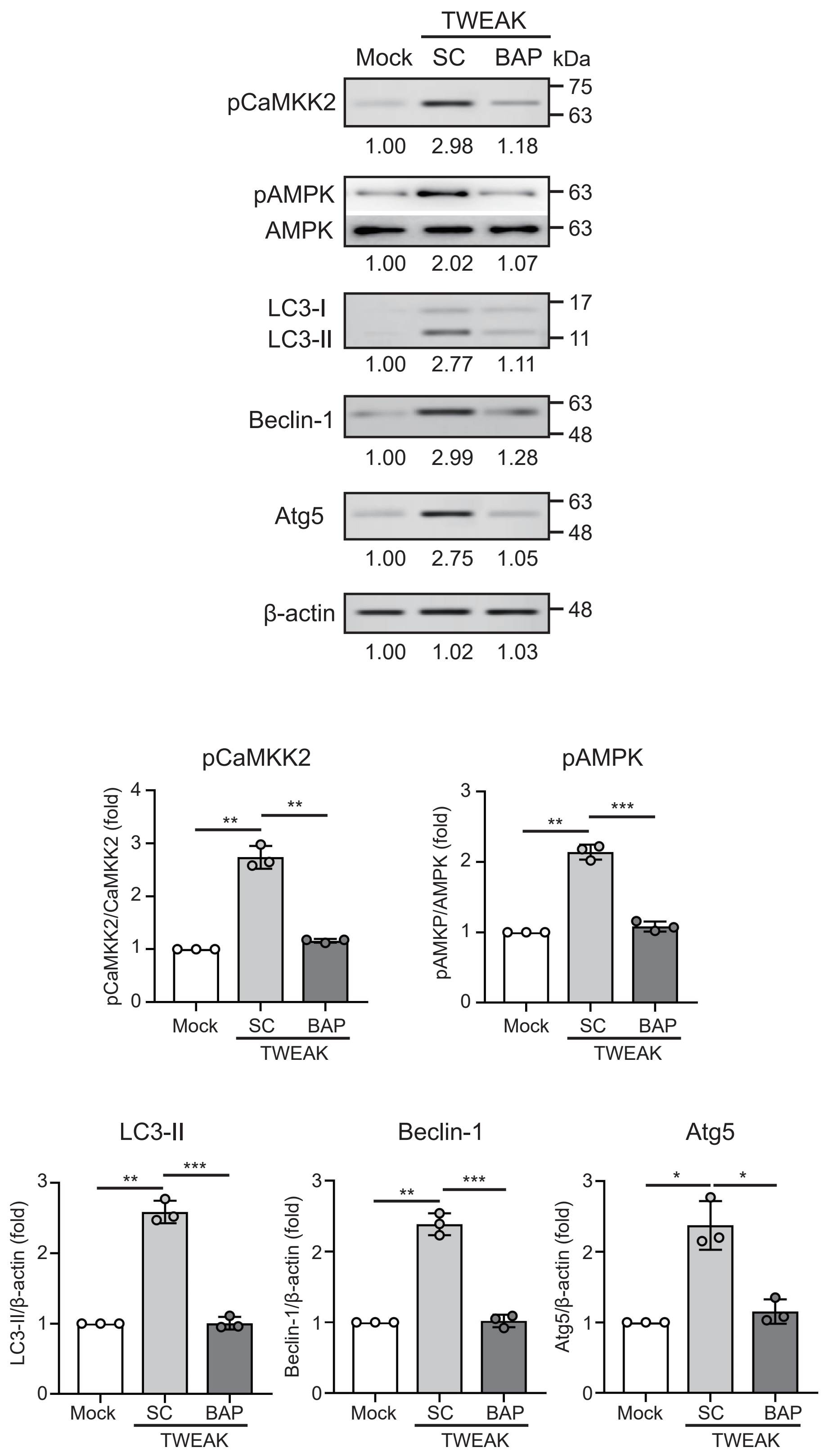
TWEAK
Mock SC BAP kDa
pCaMKK2
75
63
1.00 2.98 1.18
pAMPK
AMPK
63
63
1.00 2.02 1.07
LC3-I
LC3-II
17
11
1.00 2.77 1.11
Beclin-1
63
48
1.00 2.99 1.28
Atg5
63
48
1.00 2.75 1.05
β-actin
48
1.00 1.02 1.03
pCaMKK2
pCaMKK2/CaMKK2 (fold)
pAMPK
pAMKP/AMPK (fold)
LC3-II
LC3-II/β-actin (fold)
Beclin-1
Beclin-1/β-actin (fold)
Atg5
Atg5/β-actin (fold)
Mock SC BAP
TWEAK

Fig. 4D

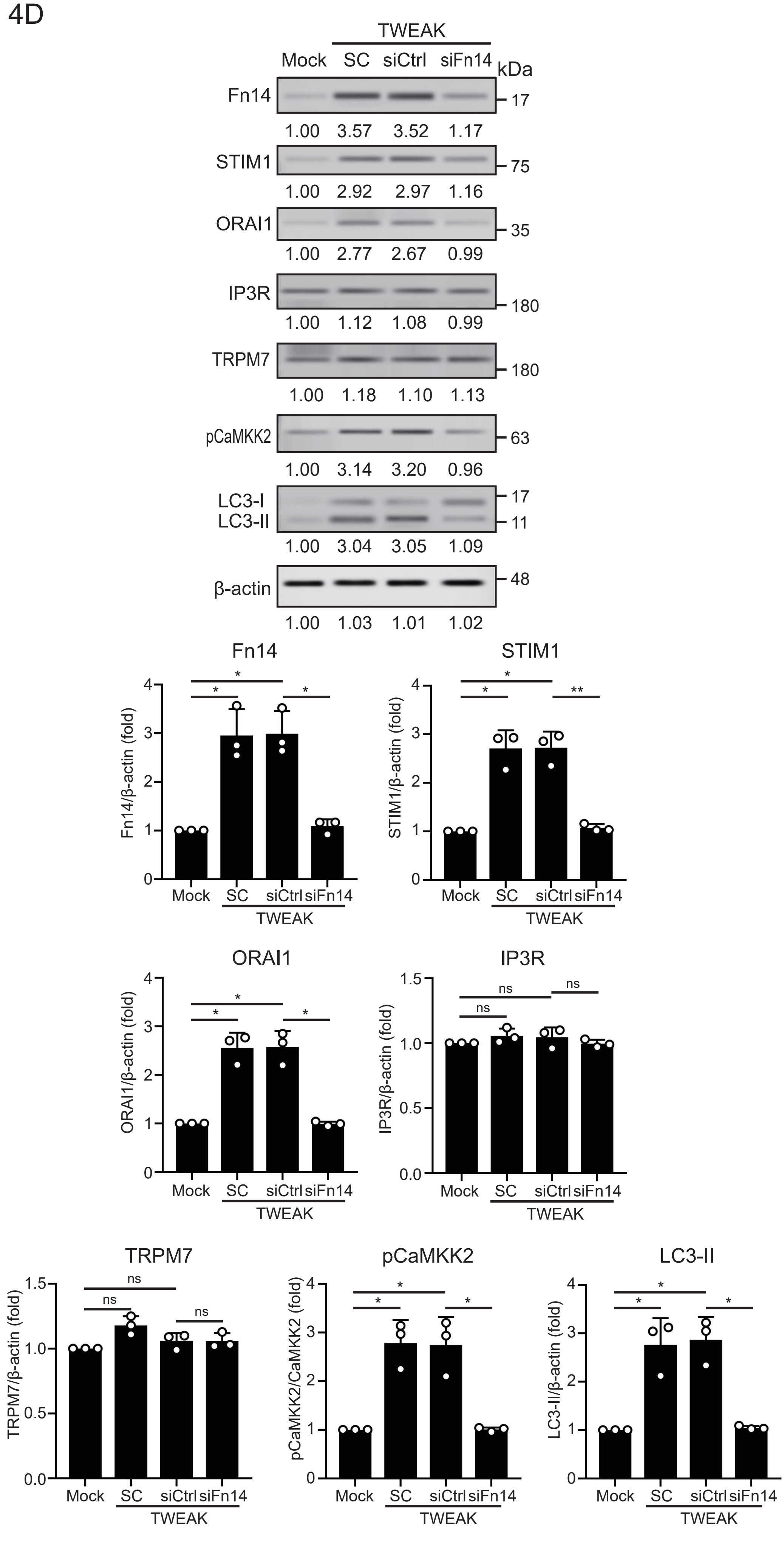

TWEAK
Mock SC siCtrl siFn14 kDa
Fn14 17
1.00 3.57 3.52 1.17
STIM1 75
1.00 2.92 2.97 1.16
ORAI1 35
1.00 2.77 2.67 0.99
IP3R 180
1.00 1.12 1.08 0.99
TRPM7 180
1.00 1.18 1.10 1.13
pCaMKK2 63
1.00 3.14 3.20 0.96
LC3-I 17
LC3-II 11
1.00 3.04 3.05 1.09
β-actin 48
1.00 1.03 1.01 1.02
Fn14
Fn14/β-actin (fold)
STIM1
STIM1/β-actin (fold)
ORAI1
ORAI1/β-actin (fold)
IP3R
IP3R/β-actin (fold)
TRPM7
TRPM7/β-actin (fold)
pCaMKK2
pCaMKK2/CaMKK2 (fold)
LC3-II
LC3-II/β-actin (fold)
Mock SC siCtrl siFn14
TWEAK
ns

Fig. 4E

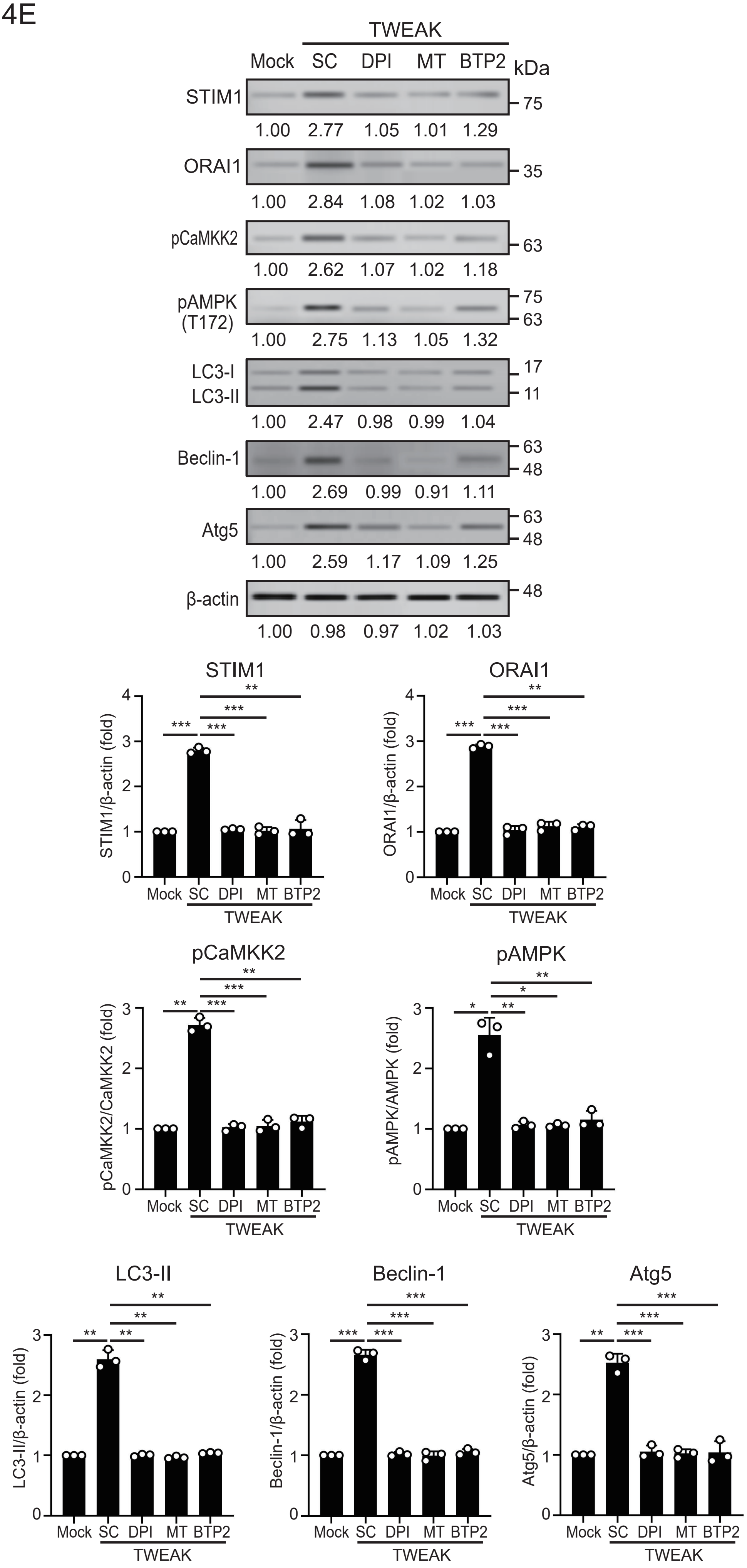
TWEAK
Mock SC DPI MT BTP2 kDa
STIM1
75
1.00 2.77 1.05 1.01 1.29
ORAI1
35
1.00 2.84 1.08 1.02 1.03
pCaMKK2
63
1.00 2.62 1.07 1.02 1.18
pAMPK (T172)
75
63
1.00 2.75 1.13 1.05 1.32
LC3-I
LC3-II
17
11
1.00 2.47 0.98 0.99 1.04
Beclin-1
63
48
1.00 2.69 0.99 0.91 1.11
Atg5
63
48
1.00 2.59 1.17 1.09 1.25
β-actin
48
1.00 0.98 0.97 1.02 1.03
STIM1
STIM1/β-actin (fold)
ORAI1
ORAI1/β-actin (fold)
pCaMKK2
pCaMKK2/CaMKK2 (fold)
pAMPK
pAMPK/AMPK (fold)
LC3-II
LC3-II/β-actin (fold)
Beclin-1
Beclin-1/β-actin (fold)
Atg5
Atg5/β-actin (fold)
Mock SC DPI MT BTP2
TWEAK

Fig. 5B

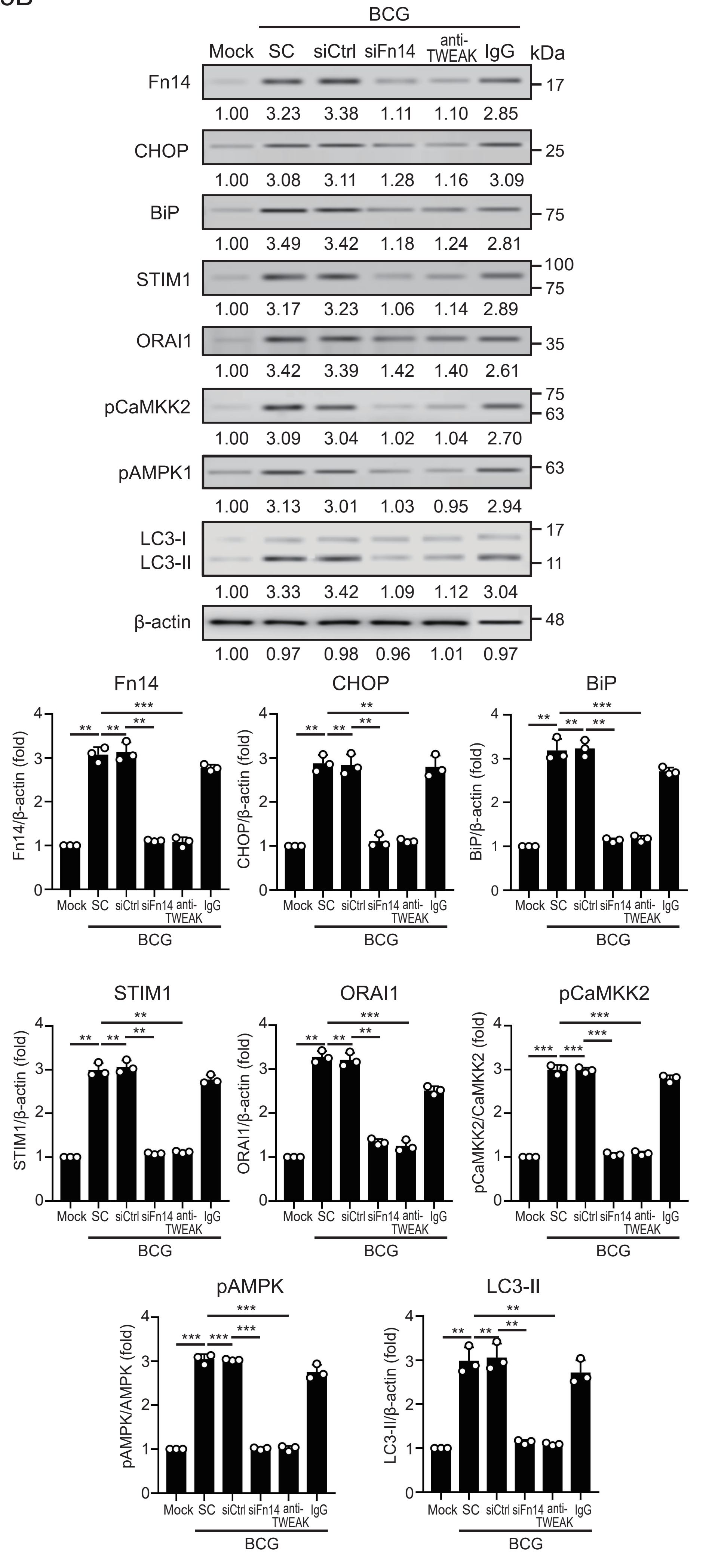
BCG
Mock SC siCtrl siFn14 anti-TWEAK IgG kDa
Fn14 17
1.00 3.23 3.38 1.11 1.10 2.85
CHOP 25
1.00 3.08 3.11 1.28 1.16 3.09
BiP 75
1.00 3.49 3.42 1.18 1.24 2.81
STIM1 100 75
1.00 3.17 3.23 1.06 1.14 2.89
ORAI1 35
1.00 3.42 3.39 1.42 1.40 2.61
pCaMKK2 75 63
1.00 3.09 3.04 1.02 1.04 2.70
pAMPK1 63
1.00 3.13 3.01 1.03 0.95 2.94
LC3-I 17
LC3-II 11
1.00 3.33 3.42 1.09 1.12 3.04
β-actin 48
1.00 0.97 0.98 0.96 1.01 0.97
Fn14
Fn14/β-actin (fold)
CHOP
CHOP/β-actin (fold)
BiP
BiP/β-actin (fold)
STIM1
STIM1/β-actin (fold)
ORAI1
ORAI1/β-actin (fold)
pCaMKK2
pCaMKK2/CaMKK2 (fold)
pAMPK
pAMPK/AMPK (fold)
LC3-II
LC3-II/β-actin (fold)
Mock SC siCtrl siFn14 anti-TWEAK IgG
BCG

Fig. 6A

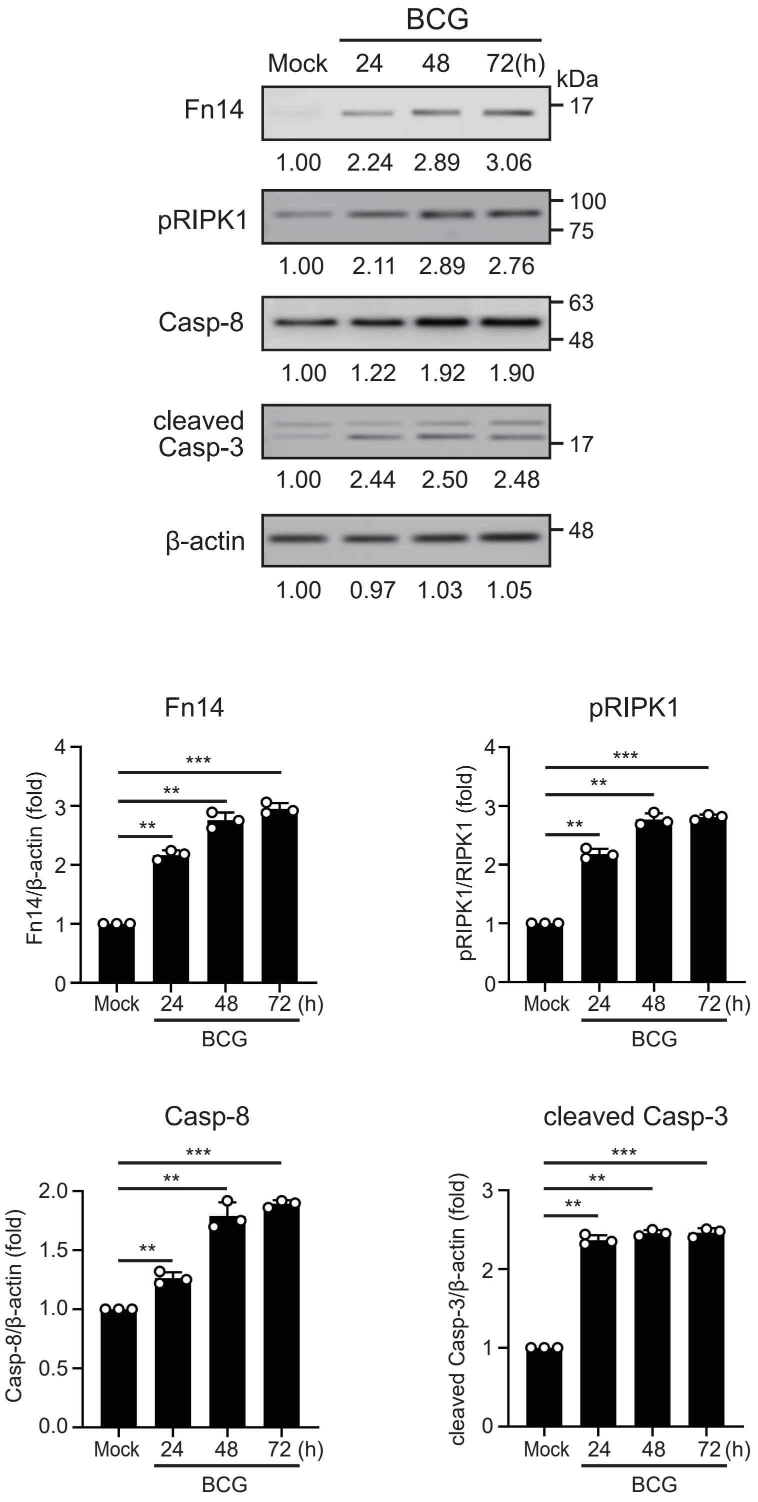
BCG
Mock 24 48 72(h)
kDa
Fn14
17
1.00 2.24 2.89 3.06
pRIPK1
100
75
1.00 2.11 2.89 2.76
Casp-8
63
48
1.00 1.22 1.92 1.90
cleaved Casp-3
17
1.00 2.44 2.50 2.48
β-actin
48
1.00 0.97 1.03 1.05
Fn14
Fn14/β-actin (fold)
pRIPK1
pRIPK1/RIPK1 (fold)
Casp-8
Casp-8/β-actin (fold)
cleaved Casp-3
cleaved Casp-3/β-actin (fold)
Mock 24 48 72 (h)
BCG

Fig. 6B

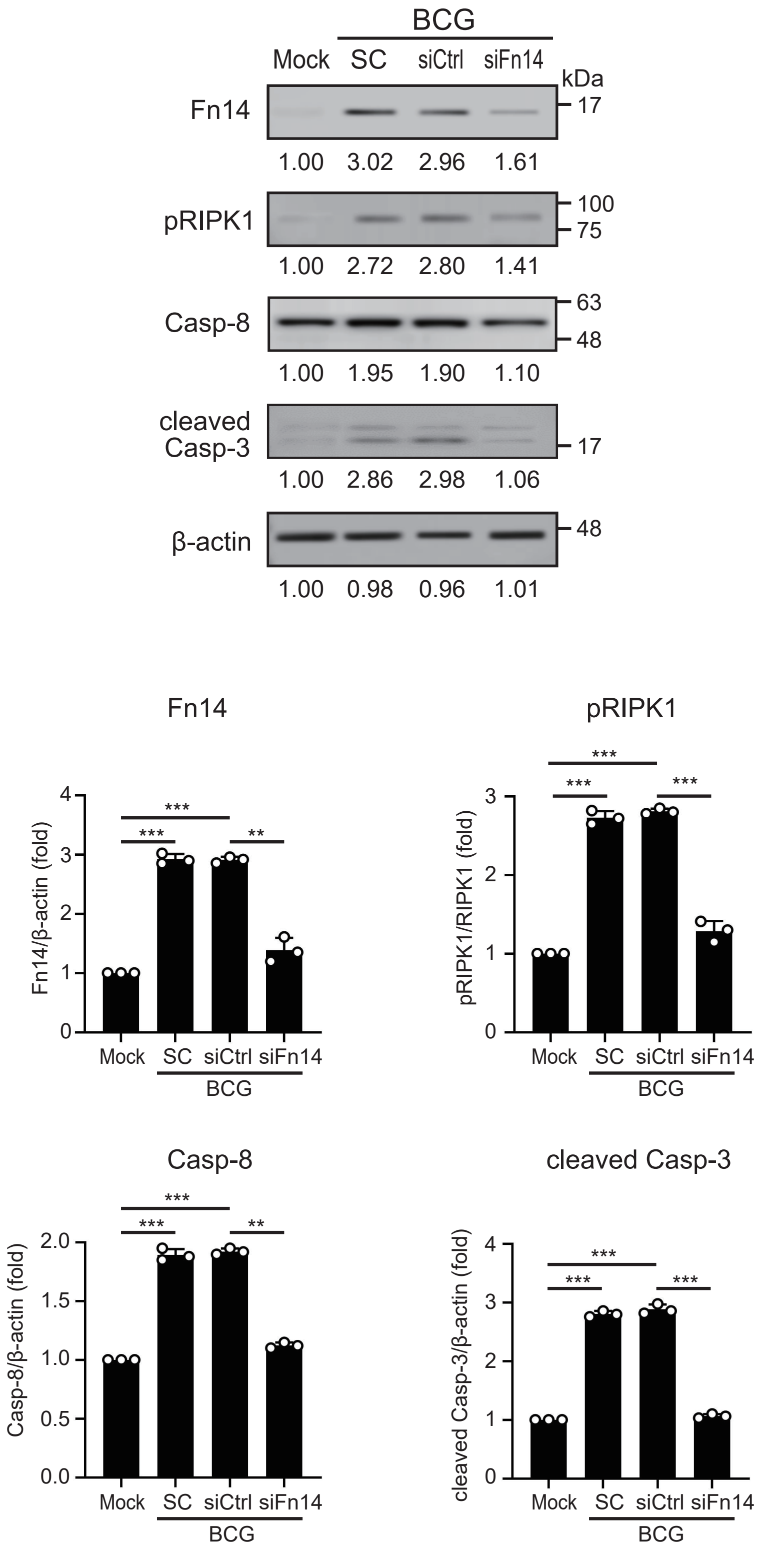
BCG
Mock SC siCtrl siFn14
kDa
Fn14
17
1.00 3.02 2.96 1.61
pRIPK1
100
75
1.00 2.72 2.80 1.41
Casp-8
63
48
1.00 1.95 1.90 1.10
cleaved Casp-3
17
1.00 2.86 2.98 1.06
β-actin
48
1.00 0.98 0.96 1.01
Fn14
Fn14/β-actin (fold)
pRIPK1
pRIPK1/RIPK1 (fold)
Casp-8
Casp-8/β-actin (fold)
cleaved Casp-3
cleaved Casp-3/β-actin (fold)
Mock SC siCtrl siFn14
BCG

Fig. 6G

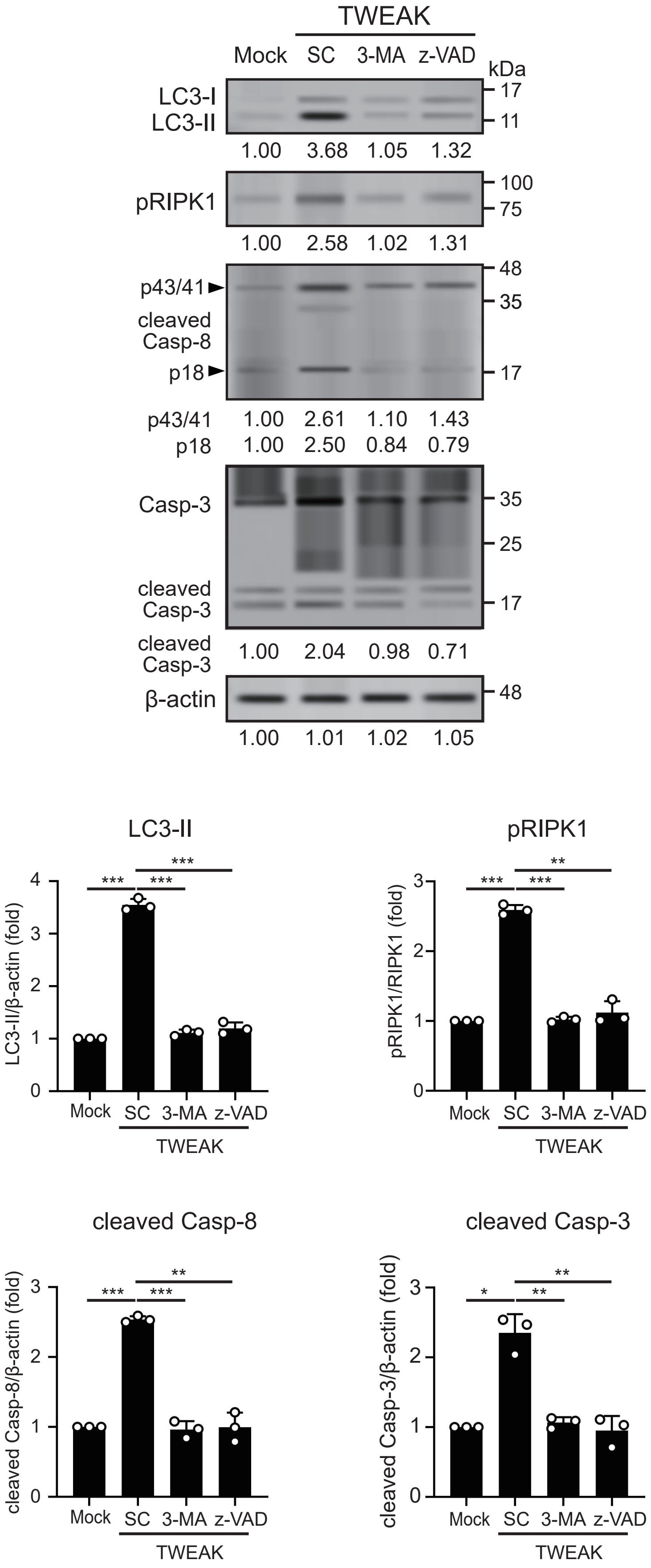
TWEAK
Mock
SC
3-MA
z-VAD
kDa
LC3-I
LC3-II
17
11
1.00 3.68 1.05 1.32
pRIPK1
100
75
1.00 2.58 1.02 1.31
p43/41
cleaved
Casp-8
p18
48
35
17
p43/41 1.00 2.61 1.10 1.43
p18 1.00 2.50 0.84 0.79
Casp-3
cleaved
Casp-3
35
25
17
cleaved
Casp-3
1.00 2.04 0.98 0.71
β-actin
48
1.00 1.01 1.02 1.05
LC3-II
LC3-II/β-actin (fold)
pRIPK1
pRIPK1/RIPK1 (fold)
cleaved Casp-8
cleaved Casp-8/β-actin (fold)
cleaved Casp-3
cleaved Casp-3/β-actin (fold)
